# Longitudinal measurement invariance in urbanization index of Chinese communities across 2000 and 2015: a Bayesian approximate measurement invariance approach

**DOI:** 10.1186/s12889-021-11691-y

**Published:** 2021-09-10

**Authors:** Ted C. T. Fong, Rainbow T. H. Ho

**Affiliations:** 1 Centre on Behavioral Health, The University of Hong Kong, 2/F, The HKJC Building for Interdisciplinary Research, 5 Sassoon Road, Pokfulam, Hong Kong, China; 2 Department of Social Work and Social Administration, The University of Hong Kong, Hong Kong, China

**Keywords:** Bayesian structural equation modeling, China health and nutrition survey, Exact invariance, Longitudinal, Psychometrics, Temporal change, Urbanization

## Abstract

**Background:**

The Urbanicity Scale was developed based on the China Health and Nutrition Survey (CHNS) to measure the urbanization index of communities according to 12 components. The present study was designed to systematically investigate the factorial validity, reliability, and longitudinal measurement invariance (LMI) of the Urbanicity Scale.

**Methods:**

Six waves of CHNS data from 2000 to 2015 were adopted. The factor structure and reliability of the Urbanicity Scale for 301 communities were examined using Bayesian exploratory factor analysis. Metric and scalar LMIs were evaluated using both the conventional exact and a novel approximate LMI approach via Bayesian structural equation modeling across various timeframes.

**Results:**

The findings verified the one-factor structure for the Urbanicity Scale, with adequate reliability. LMI was established for the Urbanicity Scale only over a shorter timeframe from 2006 to 2009 but not over a longer timeframe from 2000 to 2015. Partial LMI was found in the factor loadings and item intercepts for the Urbanicity Scale over the 2004 to 2011 period.

**Conclusion:**

Interpretation of the temporal change in urbanicity was supported only for a shorter (2006 to 2009) but not a longer timeframe (2000 to 2015). Adjustments addressing the partial non-invariance of the measurement parameters are needed for the analysis of temporal changes in urbanicity between 2004 and 2011.

**Supplementary Information:**

The online version contains supplementary material available at 10.1186/s12889-021-11691-y.

## Background

The past two decades have witnessed a remarkable social and economic transformation in mainland China [1]. Chinese societies have undergone a continuous urbanization process in which a growing population shares a modernized and improved environment for health infrastructure, housing, sanitation, communications, markets, and education [2]. Earlier research [3] relied on a crude rural/urban dichotomy and did not explicitly assess the degree of urbanization using a valid instrument. In a systematic review [4] of 11 relevant studies on urbanicity, eight of the studies did not explicitly test the psychometric properties of their urbanicity scales. Astounding inconsistencies in measurement reliability and validity point to the need for a tested, valid, and reliable measure of urbanicity. The China Health and Nutrition Survey (CHNS) [5] is a longitudinal national survey that collected 10 waves of measurements between 1989 and 2015 on societal and economic transformation at the community level and the nutrition and health status of citizens in China.

Using the CHNS data across 1989 and 2006, Jones-Smith and Popkin [6] developed an Urbanicity Scale to measure the urbanization index of communities based on 12 community-level components. Compared with scales developed in other contexts [7, 8], the scale developed by these authors was the only one rated as being high quality (rating score = 4 out of 5) in evaluations of its content validity by expert panels and reviewers, internal consistency and test–retest reliability, construct validity through exploratory factor analysis, and criterion validity. Based on the CHNS data, the Urbanicity Scale captured unique changes in community contexts across time and geographic locations and provided useful insights into associated health effects [9]. Despite the unidimensional nature of the Urbanicity Scale, no scale validation studies have been conducted with the three more recent waves of CHNS data from 2009 to 2015. Given the lack of systematic evaluation of the psychometric properties, the first objective of the present study was to examine the factorial validity and reliability of the Urbanicity Scale with CHNS data across 2000 and 2015.

Apart from factorial validity and reliability, measurement invariance across time is another essential measurement property for an assessment scale. Longitudinal measurement invariance (LMI) examines the stability of factor loadings and item intercepts and requires the scale to measure latent factors in the same way over time [10]. If LMI holds, changes in the test scores over time can be attributed to changes in the underlying construct [11]. For the CHNS data, the dynamic nature of the urbanization process often necessitates an examination of the temporal change in urbanicity [12]. To ensure meaningful comparisons of the latent factor means of urbanicity across time, the measurement structures of the Urbanicity Scale should be invariant or stable in terms of factor loadings and item intercepts.

In the case of longitudinal non-invariance of measurement parameters, temporal changes in latent factor means would be conflated with discrepancies in the loadings and/or intercepts across time [13]. This conflation would, in turn, induce measurement bias and obstruct comparisons and inferences regarding latent factor means. Despite the abundance of research on urbanization that has used the CHNS data, the assumption of LMI for the Urbanicity Scale is yet to be tested. The fulfillment of LMI would establish a psychometric basis for applied researchers to analyze temporal changes in urbanicity with other substantive variables in the CHNS data. In light of the research gap, the second objective of the present study was to examine the LMI of the Urbanicity Scale with the CHNS data across 2000 and 2015.

Conventional practice on LMI focuses on exact LMI where factor loadings and item intercepts are expected to be remain unchanged over time [14]. However, this assumption could be overly restrictive and difficult to meet [15, 16]. A recent advancement in the methodological literature [17–19] advocates the use of approximate measurement invariance via the Bayesian structural equation modeling (BSEM) approach [20]. Approximate LMI replaces the exact zero constraints on the between-time differences of the measurement parameters with approximate zero informative priors that allow some “wiggle room” [20]. The “wiggle room” permits small differences between the parameters as a compromise between zero and no constraints and facilitates comparison of the latent means. Approximate LMI has been shown to outperform exact or partial LMI in detecting the true latent mean difference [15]. The present study utilized the BSEM approach to examine the LMI of the Urbanicity Scale across 2000 and 2015 via both the exact approach and the approximate approach.

## Methods

### Study design and sample

The present study was based on community-level data originating from six waves of the CHNS in 2000, 2004, 2006, 2009, 2011, and 2015 [5]. The CHNS is a large-scale panel survey that began back in 1989 to identify potential linkages between social and economic transformation at the societal level and the nutrition and health status of citizens in China. The CHNS sampled and collected information on health, diet, nutrition, and income from individuals and households. Across the six waves of measurements from 2000 to 2015, the survey recruited a total of 301 communities via a multistage, random cluster sampling process from 12 provinces and municipal cities in China. A total of 205 sampled communities (68.1%) provided complete data across all six measurement waves and over 70% (*N* = 216, 71.8%) participated in at least four waves. Nearly one-fourth of the sampled communities (*N* = 72, 23.9%) joined the CHNS in 2011 and provided only the last two waves of data. Ethical approval for this secondary analysis was obtained from the human research ethics committee of the authors’ university (reference number = EA1809036).

### Measures

The urbanization index of the sampled communities was measured by the Urbanicity Scale developed by Jones-Smith and Popkin [6]. Urbanicity was operationalized based on 12 identified components at the community level: communications, population density, diversity, economic activity, health infrastructure, housing, traditional markets, social services, transportation, education, modern markets, and sanitation. Each of the communities was scored on these 12 components through local area administrators or official records on an 11-point anchored format from 0 to 10, with lower scores denoting lesser degrees of urbanization. The results of the original scale development study [6] suggested a unidimensional structure on urbanicity in the CHNS data. The overall urbanization index was calculated as the sum of the 12 component scores with a theoretical range from 0 to 120. The Urbanicity Scale showed good reliability (Cronbach’s *α =* 0.85–0.89) and high temporal stability in the assessments from 1989 to 2006. Urban and rural communities were classified by their baseline urbanization score in 2000, using a score of 60 as a cutoff.

### Data analysis

Table 1 displays the univariate statistics (means, standard deviations, and skewness) for the 12 components of the Urbanicity Scale. Given the lack of known multidimensionality in the Urbanicity Scale, exploratory factor analysis (EFA) was conducted using the oblique Geomin rotation in Mplus 8.4 [21] to explore one-factor to four-factor structures. Most of the 12 component scores did not deviate substantially from normal distributions, with skewness ranging from − 1.14 to 1.41 throughout the six waves of measurements. These components were modeled as continuous variables under both the frequentist and Bayesian approaches. Model comparison of the one-factor to four-factor models was performed based on the model fit and eigenvalues using parallel analysis under maximum likelihood estimation. Bayesian estimation was applied to derive the Bayesian information criterion (BIC) and posterior probabilities of the factor models via the Bayes factor. Missing data were handled via full information maximum likelihood under the missing-at-random assumption [22].

**Table 1:** Means and standard deviations of the twelve components of the Urbanicity Scale from 2000 to 2015

Item	2000	2004	2006	2009	2011	2015	Skewness
	M (SD)	M (SD)	M (SD)	M (SD)	M (SD)	M (SD)	Range
Communication	4.85 (1.18)	5.71 (1.50)	6.15 (1.43)	6.77 (1.54)	7.49 (1.56)	6.84 (1.30)	−0.73 to − 0.26
Population density	5.82 (1.40)	5.92 (1.46)	5.94 (1.47)	5.98 (1.50)	6.32 (1.56)	6.16 (1.91)	−0.37 to 0.19
Diversity	4.57 (1.17)	4.73 (1.21)	5.18 (1.28)	5.45 (1.13)	5.70 (1.29)	5.69 (1.14)	−0.02 to 0.59
Economic activity	4.71 (3.25)	5.90 (3.28)	6.53 (3.10)	6.70 (3.24)	7.57 (2.94)	7.47 (3.05)	−0.93 to 0.42
Health structure	5.64 (2.23)	5.28 (2.33)	5.03 (2.40)	5.95 (2.57)	5.92 (2.57)	5.72 (2.71)	−0.56 to 0.04
Housing	6.02 (2.67)	6.59 (2.50)	6.99 (2.33)	7.63 (2.02)	8.20 (1.77)	8.26 (1.67)	−1.14 to 0.01
Traditional market	6.00 (3.48)	5.12 (3.67)	4.86 (3.90)	4.85 (3.47)	4.87 (3.43)	5.22 (3.53)	−0.36 to 0.12
Social services	1.72 (1.10)	3.00 (2.58)	3.20 (2.74)	3.74 (3.17)	4.44 (3.30)	4.68 (3.39)	0.34 to 1.29
Transportation	5.72 (2.47)	5.91 (2.40)	5.82 (2.57)	5.97 (2.18)	4.74 (2.06)	6.16 (2.15)	−0.31 to − 0.04
Education	3.38 (1.39)	3.36 (1.38)	3.44 (1.51)	3.48 (1.46)	4.05 (1.93)	5.16 (1.47)	0.60 to 1.41
Modern market	4.76 (3.33)	4.73 (3.03)	4.60 (2.92)	4.38 (2.89)	4.62 (2.84)	4.98 (2.85)	−0.20 to 0.12
Sanitation	6.02 (3.20)	6.49 (3.02)	6.72 (2.95)	6.90 (2.90)	7.36 (2.72)	7.31 (2.36)	−0.83 to − 0.16
Total score	59.2 (18.3)	62.7 (20.4)	64.5 (20.5)	67.8 (19.6)	71.3 (19.5)	73.7 (17.6)	−0.35 to 0.04

*M* item mean, *SD* standard deviation

All Bayesian models were estimated with two Markov chain Monte Carlo chains and noninformative priors over a minimum of 10,000 iterations [23]. The latter half of the iterations was used to empirically derive the posterior parameter distribution of the parameters. Model convergence was checked using the trace plots, autocorrelation plots, and potential scale reduction criterion [24], with values of less than 1.05 implying small between-chain variation relative to within-chain variation. Under the Bayesian posterior predictive checking, an exact model fit was achieved in case of large posterior predictive *p* values (PPP > 0.10) and negative lower 95% posterior predictive limit (PPL). Approximate model fit was evaluated using the following cutoff criteria [25]: comparative fit index (CFI) ≥ 0.95 and root mean square error of approximation (RMSEA) ≤ 0.06. The reliability of the Urbanicity Scale was evaluated using Mcdonald’s omega (ω) with values of at least 0.70 and 0.80 indicating satisfactory and good composite reliability.

Following the establishment of the factorial validity and reliability, the BSEM approach was used to examine the LMI of the Urbanicity Scale from 2000 to 2015. Configural, metric, and scalar invariance models were sequentially specified to evaluate the longitudinal invariance of factor structure, factor loadings, and item intercepts, respectively [26]. First, the configural invariance model was estimated as the baseline model to allow different factor loadings and item intercepts across time. Then, exact metric and scalar invariance models were estimated that constrained the factor loadings and item intercepts to be equal across time, respectively. The comparison of nested (configural versus metric and metric versus scalar) models was made based on differences in approximate fit indices [27]. According to Chen [28], a decrease of at least 0.01 in the CFI supplemented by an increase of at least 0.015 in the RMSEA would indicate non-invariance in metric and scalar invariance models.

In the case of a lack of exact LMI, approximate LMI models were estimated under the BSEM approach [20]. This approach postulates the measurement parameters (factor loadings and item intercepts) to be approximately equal across time via zero-mean, small variance informative priors [29]. Sensitivity analysis was performed by increasing the variance (V) for the informative priors from V = 0.01 to 0.04 and 0.09, which would imply SDs of ±0.1, ±0.2, and ± 0.3 for the differences in the measurement parameters, respectively. The results of the approximate LMI models gave the time-average measurement parameters and time-specific deviations from the average value. Apart from the statistical significance of these deviations, violations in LMI were evaluated in terms of practical significance, with a relative mean change value of larger than 10% considered as indicative of substantial bias [30]. In case of substantive longitudinal non-invariance, follow-up measurement invariance tests would be conducted across different timeframes. The Mplus analysis scripts are available from the corresponding author upon reasonable request.

## Results

### Descriptive profile of the communities

The number of communities recruited across the six waves was 217, 216, 218, 218, 290, and 288, respectively, from 2000 to 2015. Rural villages accounted for 40.5% of the 301 sampled communities. Approximately one-fourth of the communities were cities (*N* = 80), one-sixth were towns (*N* = 53), and the remaining communities were suburban neighborhoods (*N* = 46). More than half (58.1%) of the recruited communities were located at rural sites and less than half (41.9%) at urban sites. The urban communities (cities or suburban neighborhoods) were significantly more urbanized (*p* < 0.001) than the rural communities (towns or rural villages) in the 2000 wave, with means (SD) of 71.7 (13.5) and 52.8 (17.1), respectively. There were significant and moderate differences (Cohen’s *d* = 0.50–0.64, *p* < 0.001) in the urbanization index between the urban and rural communities across the 2000–2015 waves.

### Factorial validity and reliability

Table 2 shows the fit indices of the Bayesian EFA models of the Urbanicity Scale from 2000 to 2015. Problems occurred in the estimation of EFA models with three and four factors with no model convergence. The two-factor model showed a better model fit than the one-factor model in terms of negative lower 95% PPL and greater PPP. However, the first factor displayed a much higher eigenvalue (5.16–6.27) than the second factor (0.90–1.14) throughout the six waves of assessments. The average eigenvalues derived from the parallel analysis ranged from 1.25 to 1.29, which consistently exceeded the eigenvalues of the second factor across the six waves. The results of the parallel analysis supported retaining only the first extracted factor. The one-factor model reliably showed a lower BIC than the two-factor model from 2000 to 2015. The results of Bayes factor testing highly favored the Urbanicity Scale being unidimensional in nature, with posterior probabilities for only one factor ranging from 0.96 to 1.00 throughout the six measurements.

**Table 2:** Fit indices of the Bayesian 1-factor and 2-factor EFA models of the Urbanicity Scale from 2000 to 2015

Year	N	Model	#	2.5% PPL	97.5% PPL	PPP	BIC	PP	RMSEA	CFI
2000	217	1-factor	36	19.4	80.8	0.002	10,277	0.9996	0.067	0.951
		2-factor	47	−17.4	53.7	0.157	10,293	0.0004	0.049	0.979
2004	216	1-factor	36	−6.9	59.4	0.052	10,428	1.0000	0.050	0.977
		2-factor	47	−22.8	41.3	0.272	10,460	0.0000	0.043	0.986
2006	218	1-factor	36	13.3	75.7	0.003	10,589	1.0000	0.063	0.963
		2-factor	47	−15.7	49.4	0.135	10,611	0.0000	0.055	0.976
2009	218	1-factor	36	16.4	79.2	0.002	10,580	1.0000	0.064	0.959
		2-factor	47	−12.7	53.1	0.119	10,603	0.0000	0.061	0.969
2011	290	1-factor	36	62.6	127.0	0.000	13,960	0.9598	0.069	0.945
		2-factor	47	14.8	81.3	0.004	13,967	0.0402	0.063	0.964
2015	288	1-factor	36	10.0	76.1	0.010	14,154	1.0000	0.054	0.962
		2-factor	47	−10.8	57.3	0.087	14,187	0.0000	0.047	0.971

*EFA* exploratory factor analysis, # number of free parameters, *PPL* posterior predictive limit, *PPP* posterior predictive *p*-value, *BIC* Bayesian information criterion, *PP* posterior probability, *RMSEA* root mean square error of approximation, *CFI* comparative fit index, *TLI* Tucker-Lewis index

In the two-factor model, there were significant and strong correlations (*r* = 0.55–0.78, *p* < 0.01) between the two factors across the six measurement waves. As shown in the supplemental table, the two-factor solution did not show a clear and consistent pattern of factor loadings on the 12 components from 2004 to 2009. There were cross-loadings or no significant loadings on a number of items, and the factor loading pattern differed substantially across the measurement waves. The unstable results pointed to potential over-extraction of the factors. The one-factor model did not show an exact fit with positive lower 95% PPL and low PPP (≤ 0.05) but displayed an approximate fit to the data from 2000 to 2015 in terms of RMSEA and CFI. All 12 components loaded significantly and substantially (*λ* = 0.41–0.88, *p* < 0.01) on the total urbanicity factor and the one-factor solution from 2004 to 2009 (as displayed in the supplemental table). In the one-factor model, the urbanicity factor exhibited good levels of composite reliability (ω = 0.88, 0.91, 0.91, 0.90, 0.90, and 0.85) across the six waves of measurements from 2000 to 2015, respectively.

### Measurement invariance across 2000–2015

Table 3 shows the fit indices of various one-factor BSEM measurement invariance models for the Urbanicity Scale across different timeframes. Under noninformative priors, the configural model displayed adequate approximate fit (CFI = 1.00 and RMSEA = 0.000) to the 2000–2015 data. Specification of exact metric invariance led to greatly increased PPLs and substantial deteriorations in the fit indices (ΔCFI = − 0.025 and ΔRMSEA = + 0.024) compared with the configural model, implying that the assumption of exact metric invariance was untenable. The alternative specification of approximate metric invariance with V = 0.01 did not result in substantial decrements in the fit indices (ΔCFI = − 0.006 and ΔRMSEA = + 0.012). As shown in Table 4, seven of the 12 components (communications, population density, diversity, housing, social services, education, and sanitation) displayed significant and substantial (Δ = 17.5–42.7%) deviations from the average λ across time. The majority (10/13) of the greatest deviations from the average λ were located in either the 2000 or the 2015 wave.

**Table 3:** Fit indices of 1-factor BSEM measurement invariance models for the Urbanicity Scale

Model specification	Prior variance	pD	2.5% PPL	97.5% PPL	RMSEA	CFI	ΔRMSEA	ΔCFI
2000–2015 (*N* = 301):								
**Configural**	**/**	**389**	**631.5**	**1101.6**	**0.000**	**1.000**	**/**	**/**
Metric	Exact	309	1123.6	1584.5	0.024	0.975	+ 0.024	−0.025
**Metric**	**V = 0.01**	**323**	**819.4**	**1276.3**	**0.012**	**0.994**	**+ 0.012**	**− 0.006**
Scalar	Exact	268	2505.7	3004.3	0.050	0.887	+ 0.038	− 0.107
Scalar	V = 0.01	280	1791.9	2250.2	0.039	0.933	+ 0.027	−0.061
Scalar	V = 0.04	302	1070.4	1518.8	0.022	0.978	+ 0.010	−0.016
**Scalar**	**V = 0.09**	**311**	**897.6**	**1350.6**	**0.016**	**0.989**	**+ 0.004**	**−0.005**
2004–2011 (*N* = 295):								
**Configural**	**/**	**214**	**264.0**	**564.1**	**0.012**	**0.996**	**/**	**/**
Metric	Exact	173	450.5	732.2	0.027	0.979	+ 0.015	−0.017
**Metric**	**V = 0.01**	**181**	**329.5**	**619.5**	**0.018**	**0.990**	**+ 0.006**	**−0.006**
Scalar	Exact	144	1062.4	1368.9	0.052	0.920	+ 0.034	−0.070
Scalar	V = 0.01	160	580.9	869.3	0.034	0.966	+ 0.016	−0.024
**Scalar**	**V = 0.04**	**171**	**380.3**	**674.0**	**0.023**	**0.985**	**+ 0.005**	**−0.005**
2006–2009 (*N* = 220):								
**Configural**	**/**	**84**	**41.6**	**170.5**	**0.046**	**0.972**	**/**	**/**
**Metric**	**Exact**	**72**	**66.9**	**192.1**	**0.050**	**0.965**	**+ 0.004**	**−0.007**
Scalar	Exact	61	220.9	339.1	0.071	0.928	+ 0.021	−0.037
**Scalar**	**V = 0.01**	**66**	**103.2**	**229.1**	**0.056**	**0.956**	**+ 0.006**	**−0.009**

*PPL* posterior predictive limit, *pD* Estimated number of parameters, *RMSEA* root mean square error of approximation, *CFI* comparative fit index; Δ = change in fit index over the previous nested (bolded) model

**Table 4:** Results of approximate metric invariance model with prior variance of 0.01 for the differences in factor loadings of the Urbanicity Scale from 2000 to 2015

Item	λ		Deviations from the average λ						Δ
	Average	SD	2000	2004	2006	2009	2011	2015	
Communication	0.89	0.05	−0.09*	0.06	0.01	0.04	0.11*	−0.14*	28.0%
Population density	1.02	0.07	−0.11*	− 0.02	− 0.04	0.02	0.00	0.16*	26.1%
Diversity	0.71	0.05	−0.02	0.06*	0.07*	− 0.03	0.04	−0.13*	27.7%
Economic activity	2.13	0.11	0.01	0.03	−0.03	0.04	−0.03	− 0.02	3.2%
Health structure	1.09	0.08	−0.01	0.06	0.06	−0.00	−0.06	− 0.05	11.1%
Housing	1.54	0.08	0.16*	0.12*	0.07	−0.01	− 0.11*	−0.24*	26.5%
Traditional market	1.65	0.13	−0.05	0.02	0.06	0.04	−0.01	−0.06	6.9%
Social services	0.92	0.07	−0.30*	0.09	0.04	0.03	0.08	0.07	42.7%
Transportation	0.96	0.08	0.04	−0.02	0.02	− 0.01	−0.00	− 0.03	6.8%
Education	1.10	0.07	−0.08*	−0.08*	0.06*	0.02	0.19*	−0.11*	26.6%
Modern market	1.84	0.11	−0.02	0.03	0.02	−0.02	0.03	−0.03	3.6%
Sanitation	2.03	0.11	0.12*	−0.00	0.05	0.04	0.03	−0.24*	17.5%

* *p* < 0.05; λ = factor loading; *SD* standard deviation; Δ = maximum proportional deviation from the average loading across the 2000–2015 waves. The top two significant deviations from the average loading are highlighted in bold

In terms of scalar longitudinal invariance, specification of exact scalar invariance led to greatly increased PPLs and substantial deteriorations in the fit indices (ΔCFI = − 0.107 and ΔRMSEA = + 0.038) relative to the approximate metric invariance model. This rejected the assumption of exact scalar invariance from 2000 to 2015. The alternative specification of approximate scalar invariance with V = 0.01 and V = 0.04 still resulted in substantial decrements in the fit indices (ΔCFI = − 0.016 to − 0.061 and ΔRMSEA = + 0.010 to + 0.027). A further increase of prior variance to V = 0.09 led to a comparable model fit (ΔCFI = − 0.005 and ΔRMSEA = + 0.004). As Table 5 indicates, significant deviations were found across time from the average ν for all 12 components. Ten of the 12 deviations were considered substantial (Δ = 10.4–60.2%); the exceptions were those for population density (3.8%) and modern markets (8.0%). Over half (15/24) of the greatest deviations from the average ν occurred in either the 2000 or the 2015 wave.

**Table 5:** Results of approximate scalar invariance model with prior variance of 0.09 for the differences in item intercepts of the Urbanicity Scale from 2000 to 2015

Item	ν		Deviations from the average ν						Δ
	Average	SD	2000	2004	2006	2009	2011	2015	
Communication	6.46	0.06	−1.31*	− 0.45*	− 0.07	0.51*	0.97*	0.36*	35.3%
Population density	6.26	0.09	− 0.13*	−0.03	0.01	0.08*	0.11*	− 0.04	3.8%
Diversity	5.36	0.06	−0.58*	− 0.40*	0.03	0.25*	0.34*	0.36*	17.4%
Economic activity	6.91	0.15	−1.05*	−0.32*	0.13	0.21*	0.55*	0.49*	23.1%
Health structure	5.75	0.10	0.13	−0.12	−0.35*	0.25*	0.12	−0.03	10.4%
Housing	7.66	0.10	−0.94*	−0.46*	− 0.15*	0.36*	0.57*	0.61*	20.2%
Traditional market	5.39	0.15	0.51*	−0.01	−0.12	− 0.15	−0.25*	0.02	14.2%
Social services	3.42	0.10	−1.37*	−0.15	0.00	0.30*	0.52*	0.69*	60.2%
Transportation	5.81	0.10	0.05	0.18	0.11	0.22*	−0.90*	0.33*	21.2%
Education	4.13	0.09	−0.33*	−0.32*	− 0.22*	− 0.19*	0.00	1.07*	34.0%
Modern market	4.89	0.14	0.09	0.15	−0.01	− 0.23*	− 0.16*	0.16*	8.0%
Sanitation	7.19	0.14	−0.51*	− 0.12	0.05	0.18*	0.22*	0.19*	10.2%

* *p* < 0.05; ν = item intercept; *SD* standard deviation; Δ = maximum proportional deviation from the average intercept across the 2000–2015 waves. The top two significant deviations from the average intercept are highlighted in bold

### Measurement invariance across 2004–2011

The previous results identified the non-invariant factor loadings and item intercepts as occurring mostly in the 2000 or 2015 waves. As a result, follow-up measurement invariance tests were conducted for the Urbanicity Scale across 2004 and 2011. As shown in Table 3, the configural model displayed an adequate approximate fit (CFI = 0.996 and RMSEA = 0.012) to the 2004–2011 data. Specification of exact metric invariance led to greatly increased PPLs and substantial deteriorations in the fit indices (ΔCFI = − 0.017 and ΔRMSEA = + 0.015) relative to the configural model, implying the untenability of exact metric invariance. Specification of approximate metric invariance with V = 0.01 resulted in a fit (ΔCFI = − 0.006 and ΔRMSEA = + 0.006) comparable to that of the configural model. As shown in Table 6, three of the 12 components (diversity, housing, and education) showed significant and substantial (Δ = 12.1–22.2%) deviations from the average λ across time. The remaining nine components did not display significant and substantial (Δ = 2.0–10.3%) deviations from the average λ across time. The majority (4/5) of the greatest deviations from the average λ were found in either the 2004 or the 2011 wave.

**Table 6:** Results of approximate metric invariance model with prior variance of 0.01 for the differences in factor loadings of the Urbanicity Scale from 2004 to 2011

Item	λ		Deviations from the average λ				Δ
	Average	SD	2004	2006	2009	2011	
Communication	1.12	0.07	0.01	− 0.04	− 0.01	0.04	7.4%
Population density	1.05	0.08	−0.02	− 0.04	0.02	0.04	7.0%
Diversity	0.86	0.06	0.03	0.04	−0.06*	− 0.01	12.1%
Economic activity	2.31	0.13	0.02	−0.02	0.04	−0.04	3.6%
Health structure	1.28	0.11	0.05	0.05	−0.02	−0.08	10.3%
Housing	1.75	0.09	0.17*	0.08*	−0.05	− 0.20*	21.0%
Traditional market	2.07	0.16	−0.01	0.04	0.01	−0.04	3.9%
Social services	1.66	0.12	0.01	−0.03	0.01	0.02	3.0%
Transportation	1.06	0.10	−0.01	0.03	0.00	−0.01	3.6%
Education	1.27	0.09	−0.14*	0.02	−0.03	0.15*	22.2%
Modern market	2.11	0.13	0.02	0.01	−0.03	0.00	2.0%
Sanitation	2.35	0.13	0.02	0.04	0.02	−0.08	4.9%

* *p* < 0.05; λ = factor loading; *SD* standard deviation; Δ = maximum proportional deviation from the average loading across the 2004–2011 waves. The top two significant deviations from the average loading are highlighted in bold

In terms of scalar invariance, specification of exact scalar invariance led to sharply increased PPLs and substantial deteriorations in the fit indices (ΔCFI = − 0.070 and ΔRMSEA = + 0.034) relative to the approximate metric invariance model. This rejected the assumption of exact scalar invariance from 2004 to 2011. Although specification of approximate scalar invariance with V = 0.01 resulted in substantial decrements in the fit indices (ΔCFI = − 0.024 and ΔRMSEA = + 0.016), a further increase of prior variance to V = 0.04 led to a model fit (ΔCFI = − 0.005 and ΔRMSEA = + 0.005) comparable to that of the approximate metric invariance model. As can be observed in Table 7, significant deviations were found across time from the average ν for 10 of the 12 components (not for traditional and modern markets). Five of the 10 deviations (in communications, diversity, housing, social services, and transportation) were considered substantial (Δ = 12.2–18.6%), and the others were considered non-substantial (Δ = 2.0–9.1%). The majority (16/19) of the greatest deviations from the average ν occurred in either the 2004 or the 2011 wave.

**Table 7:** Results of approximate scalar invariance model with prior variance of 0.04 for the differences in item intercepts of the Urbanicity Scale from 2004 to 2011

Item	ν		Deviations from the average ν				Δ
	Average	SD	2004	2006	2009	2011	
Communication	6.79	0.09	−0.61*	− 0.28*	0.24*	0.65*	18.6%
Population density	6.33	0.10	−0.07*	− 0.03	0.04	0.06*	2.0%
Diversity	5.45	0.07	−0.44*	−0.02	0.18*	0.27*	13.0%
Economic activity	7.24	0.18	−0.35*	−0.01	0.05	0.31*	9.1%
Health structure	5.75	0.12	−0.04	− 0.24*	0.21*	0.08	7.8%
Housing	7.80	0.13	−0.53*	−0.21*	0.28*	0.46*	12.6%
Traditional market	5.22	0.19	0.11	0.01	−0.01	−0.11	4.3%
Social services	3.93	0.15	−0.24*	−0.11	0.12	0.24*	12.2%
Transportation	5.66	0.11	0.20*	0.15	0.29*	−0.64*	16.4%
Education	3.92	0.11	−0.15*	−0.03	− 0.01	0.19*	8.6%
Modern market	4.92	0.16	0.16	0.06	−0.10	− 0.11	5.4%
Sanitation	7.36	0.17	−0.18*	−0.02	0.10	0.11	3.9%

* *p* < 0.05; ν = item intercept; *SD* standard deviation; Δ = maximum proportional deviation from the average intercept across the 2004–2011 waves. The top two significant deviations from the average intercept are highlighted in bold

### Measurement invariance across 2006–2009

The previous results identified most non-invariant factor loadings and item intercepts as occurring in the 2004 or 2011 wave. Follow-up measurement invariance tests were conducted for the Urbanicity Scale across 2006 and 2009. As was shown in Table 3, the configural model displayed adequate approximate fit (CFI = 0.972 and RMSEA = 0.046) to the 2006–2009 data. Specification of exact metric invariance led to a fit comparable to that of the configural model without substantial deteriorations in the fit indices (ΔCFI = − 0.007 and ΔRMSEA = + 0.004). Specification of exact scalar invariance led to increased PPLs and substantial deteriorations in the fit indices (ΔCFI = − 0.037 and ΔRMSEA = + 0.021) relative to the exact metric invariance model. These results supported the assumption of exact metric invariance but not exact scalar invariance from 2006 to 2009. Specification of approximate scalar invariance with V = 0.01 led to a model fit (ΔCFI = − 0.009 and ΔRMSEA = + 0.006) comparable to that for the exact metric invariance model. According to Table 8, significant deviations were found across time from the average ν for four of the 12 components (communications, diversity, health infrastructure, and housing). None of these four deviations were considered substantial (Δ = 3.3–5.6%).

**Table 8:** Results of approximate scalar invariance model with prior variance of 0.01 for the differences in item intercepts of the Urbanicity Scale from 2006 to 2009

Item	ν		Deviations from the average ν		Δ
	Average	SD	2006	2009	
Communication	6.44	0.09	−0.16*	0.16*	5.1%
Population density	5.97	0.10	−0.02	0.02	0.6%
Diversity	5.33	0.08	−0.09*	0.09*	3.3%
Economic activity	6.64	0.20	−0.01	0.01	0.4%
Health structure	5.41	0.15	−0.11*	0.11*	4.1%
Housing	7.35	0.15	−0.21*	0.21*	5.6%
Traditional market	4.88	0.22	0.01	−0.01	0.2%
Social services	3.45	0.17	−0.04	0.04	2.3%
Transportation	5.91	0.14	−0.01	0.01	0.5%
Education	3.47	0.10	−0.01	0.01	0.7%
Modern market	4.51	0.19	0.04	−0.04	1.7%
Sanitation	6.83	0.20	−0.04	0.04	1.1%

* *p* < 0.05; ν = item intercept; *SD* standard deviation; Δ = maximum proportional deviation from the average intercept across the 2006–2009 waves. The significant deviations from the average intercept are highlighted in bold

## Discussion

The present study involved a systematic evaluation of the psychometric properties of the Urbanicity Scale using six waves of CHNS data spanning from 2000 to 2015. In terms of dimensionality, the EFA results obtained from both the frequentist approach (eigenvalues via parallel analysis) and the Bayesian approach (BIC and posterior probabilities) support the one-factor structure as a parsimonious fit of the underlying construct of urbanicity. Despite the better model fit, the two-factor model is less parsimonious, less interpretable, and subject to potential factor over-extraction. These findings corroborate the unidimensional nature of the Urbanicity Scale. Good omega coefficients were consistently found for the total urbanicity factor throughout all six waves of measurements, suggesting adequate reliability for the Urbanicity Scale.

This study is the first to systematically investigate the LMI for the Urbanicity Scale across a 15-year period using exact and approximate LMI approaches via BSEM. Across six waves of measurements from 2000 to 2015, exact LMI was rejected for both metric invariance (factor loadings) and scalar invariance (item intercepts). Approximate LMI models resulted in adequate model fits with prior variances specified for the differences in factor loadings (V = 0.01) and item intercepts (V = 0.09). However, among the 12 items, statistically significant (*p* < 0.05) and practically substantial (Δ > 10%) deviations were found in seven factor loadings and 10 item intercepts across time. The occurrence of non-invariance in the majority of instances for both measurement parameters essentially implies a lack of LMI for the scale across this timeframe. In particular, the 2000 wave of CHNS data exhibited the greatest degrees of non-invariance. This discrepancy could reflect potential alterations or shifts in the scoring algorithm of the components in the Urbanicity Scale between the 2000 and 2004 waves.

Given the lack of LMI across 2000 and 2015, follow-up LMI analyses were conducted of the scale over shorter timeframes. Across four waves of measures from 2004 to 2011, exact LMI was not supported in either factor loadings or item intercepts. Approximate LMI models resulted in adequate model fits using zero-mean, small variance informative priors for the differences in factor loadings (V = 0.01) and item intercepts (V = 0.04). For the 12 items, statistically significant and practically substantial deviations were found in three factor loadings and five item intercepts across time, and two components (diversity and housing) displayed substantial non-invariance in both parameters. Further investigation of LMI supported the existence of exact metric invariance across the two waves from 2006 to 2009. Although the Urbanicity Scale displayed approximate but not exact scalar invariance across 2006 and 2009, statistically significant deviations were found in only four item intercepts, with none being practically substantial (Δ < 6%). These results demonstrate LMI for the Urbanicity Scale across the timeframe from 2006 to 2009.

### Practical implications

The present study revealed intriguing findings regarding the measurement stability of the Urbanicity Scale, with the degrees of non-invariance increasing in proportion to the length of the timeframe being scrutinized. Our findings did not support any form of (exact or approximate) LMI across the longest timespan from 2000 to 2015. The substantial degrees of non-invariance in both factor loadings and item intercepts (Tables 4 and 5) imply that no partial LMI was feasible under this timeframe. The findings demonstrated longitudinal measurement non-invariance from 2000 to 2015, and comparisons of the latent means of urbanicity would probably be confounded by the existing measurement biases across time. The lack of psychometric support suggests that future longitudinal studies using the CHNS should not analyze temporal changes in urbanicity across such a long time span.

The present study did, however, demonstrate LMI of the scale across adjacent waves between 2006 and 2009. The trivial non-invariance in the item intercepts (Table 8) should not have substantial impacts on inferences of temporal changes in urbanicity. A psychometric basis was established for meaningful comparisons of the latent means of urbanicity across this timeframe. Our findings appear to point toward partial approximate LMI for the Urbanicity Scale across 2004 and 2011. Comparisons of the latent means of urbanicity across 2004 and 2011 are theoretically plausible through specification of partial invariance models. However, the associated temporal changes in urbanicity should be interpreted with caution, and future researchers need to properly adjust for the partial non-invariance of the measurement parameters in the scale.

### Methodological implications

Assessing the LMI of a measurement scale is fundamental to establish the temporal stability of the assessed constructs and thus enable meaningful interpretation of longitudinal findings [31]. Nevertheless, examination of the LMI of assessment scales over multiple (six) repeated measurements remains relatively rare. The present study contributes to the literature on urbanicity through its novel application of the approximate measurement invariance approach. The approximate LMI approach allows researchers to make unequivocal trade-offs between the degrees of model fit and measurement non-invariance across time [15]. This approach is useful in assisting researchers to obtain a balance between achieving a well-fitting model, adhering to the invariance requirements, and making comparisons possible [32]. In addition, the use of informative priors via BSEM helps researchers evaluate the statistical and practical significance of between-time differences among measurement parameters. Given the frequent rejection of classical LMI tests in applied research, the approximate LMI approach could be regarded as a promising and realistic alternative.

Apart from the Bayesian approximate measurement invariance approach, an alignment method was proposed by Muthén and Asparouhov [33] for multiple-group confirmatory factor analysis. Their alignment method has the capability to estimate group-specific factor means and variances in factor models without requiring exact measurement invariance. The ability to compute aligned factor scores for the full sample despite the presence of non-invariance in some groups facilitates comparisons of factor means on the basis of a configural invariance model [34]. This technique is suitable and feasible for assessing measurement invariance in large data sets across numerous groups, such as in comparisons across multiple countries. Munck, Barber, and Torney-Purta [35] recently demonstrated the usefulness of the alignment method for group comparisons of European youth attitudes toward immigrants across a total of 92 groups (country by cohort by gender). Future studies are recommended to evaluate the use of the alignment method in longitudinal measurement invariance and the possibility of integrating model alignment with approximate measurement invariance via the Bayesian approach.

### Study limitations

Several limitations of the present study should be pointed out. First, measurement invariance of the Urbanicity Scale was only evaluated across time and not across community context (rural vs. urban sites). The relatively small sample sizes (*N* < 200) for the rural and urban sites did not provide adequate statistical power for accurate detection of scale measurement invariance. Additional studies are required to elucidate potential measurement biases related to contextual factors such as geographic locations and contexts. Second, the CHNS did not include data on community characteristics such as social networks and culture. The Urbanicity Scale derived from the CHNS data could place a disproportionately large emphasis on economic activities, which would raise doubts regarding the content validity and item coverage of the scale. Third, the present study focused only on the factorial validity and LMI but not the convergent validity and divergent validity of the Urbanicity Scale. Investigation of its convergent and divergent validity with reference to individual-level outcomes such as obesity, physical activity, and lifestyle would require multilevel analyses that were outside the scope of the present study. Future research should attempt to evaluate the associations between urbanicity and these substantive variables as in previous studies [9, 12] while taking into account the measurement non-invariance in the parameters across measurement waves.

## Conclusions

The present study contributed a systematic evaluation of the factorial validity, reliability, and LMI of the Urbanicity Scale using the BSEM approach with six waves of CHNS data from 2000 to 2015. The findings verified the one-factor structure of the Urbanicity Scale with adequate reliability. Regarding measurement invariance across time, LMI was only established for the Urbanicity Scale over a shorter timeframe from 2006 to 2009 and not over a longer timeframe from 2000 to 2015. Interpretations of temporal changes in urbanicity are recommended only for the former timeframe. Analyzing the temporal change in urbanicity from 2004 to 2011 requires proper adjustments for the partial non-invariance of the measurement parameters.

## Supplementary Information

**Additional file 1.**

**Additional file 2.**

**Additional file 3.**

**Additional file 4.**

## Data Availability

All data generated or analysed during this study are included in this published article as a supplementary information file.

## Abbreviations

CHNS: China Health and Nutrition Survey
LMI: Longitudinal measurement invariance
BSEM: Bayesian structural equation modeling
EFA: Exploratory factor analysis
BIC: Bayesian information criterion
PPP: Posterior predictive *p* values
PPL: Posterior predictive limit
CFI: Comparative fit index
RMSEA: Root mean square error of approximation
