# Supplementary material for: Longitudinal measurement invariance in urbanization index of Chinese communities across 2000 and 2015: a Bayesian approximate measurement invariance approach
Source: BMC Public Health. 2021 Sep 10;21:1653. doi: 10.1186/s12889-021-11691-y (PMC8431910; doi:10.1186/s12889-021-11691-y)
Supplement: Supplementary file 2 — Additional file 2. [file 12889_2021_11691_MOESM2_ESM.doc]

| **Table S1.** Factor structure of the 1-factor and 2-factor models of the Urbanicity Scale in the 2004 – 2009 waves | | | | | | | | | | | |
| --- | --- | --- | --- | --- | --- | --- | --- | --- | --- | --- | --- |
|  | 2004 wave | | |  | 2006 wave | | |  | 2009 wave | | |
|  | 1-factor | 2-factor | |  | 1-factor | 2-factor | |  | 1-factor | 2-factor | |
| Communication | 0.74* | **0.48** | **0.32** |  | 0.74* | **0.42*** | **0.36*** |  | 0.69* | 0.78* | -0.01 |
| Population density | 0.61* | **0.39** | **0.27** |  | 0.57* | 0.48* | 0.10 |  | 0.60* | 0.51* | 0.12 |
| Diversity | 0.75* | 0.55* | 0.24 |  | 0.72* | 0.59* | 0.15 |  | 0.66* | 0.39* | 0.31 |
| Economic activity | 0.74* | 0.67* | 0.07 |  | 0.72* | 0.55* | 0.20 |  | 0.78* | 0.01 | 0.81* |
| Health structure | 0.65* | **0.39** | **0.31** |  | 0.64* | **0.34*** | **0.34*** |  | 0.45* | 0.02 | 0.44* |
| Housing | 0.88* | 0.90* | -0.01 |  | 0.88* | 0.87* | 0.02 |  | 0.87* | **0.41*** | **0.51*** |
| Traditional market | 0.57* | 0.01 | 0.58* |  | 0.63* | -0.01 | 0.74* |  | 0.60* | 0.56* | 0.06 |
| Social services | 0.65* | **0.42** | **0.27** |  | 0.53* | 0.48* | 0.05 |  | 0.54* | 0.53* | 0.02 |
| Transportation | 0.41* | 0.05 | 0.41* |  | 0.44* | **0.26** | **0.20** |  | 0.47* | 0.33* | 0.17 |
| Education | 0.73* | 0.71* | 0.00 |  | 0.75* | 0.73* | 0.01 |  | 0.74* | 0.51* | 0.26 |
| Modern market | 0.74* | **0.35** | **0.47** |  | 0.78* | 0.13 | 0.74* |  | 0.76* | 0.55* | 0.26 |
| Sanitation | 0.82* | 0.83* | -0.01 |  | 0.85* | 0.90* | -0.02 |  | 0.85* | 0.01 | 0.88* |
| *Note. Bolded values denote double factor loadings or no significant loadings in the 2-factor model.* | | | | | | | | | | | |
