# Supplementary material for: Longitudinal measurement invariance in urbanization index of Chinese communities across 2000 and 2015: a Bayesian approximate measurement invariance approach
Source: BMC Public Health. 2021 Sep 10;21:1653. doi: 10.1186/s12889-021-11691-y (PMC8431910; doi:10.1186/s12889-021-11691-y)
Supplement: Supplementary file 3 — Additional file 3. [file 12889_2021_11691_MOESM3_ESM.pdf]

**CHINA ECONOMIC, POPULATION, NUTRITION, AND HEALTH SURVEY**

**2004 COMMUNITY QUESTIONNAIRE**

Province: 21 Liaoning 23 Heilongjiang 32 Jiangsu 37 Shandong 41 Henan ☐☐T1
42 Hubei 43 Hunan 45 Guangxi 52 Guizhou

Urban Site: 1 | Rural Site: 2 ☐T2

City: ___________
1 First city
2 Second city

County: ____________ ☐T3
1 First county
2 Second county
3 Third county
4 Fourth county

Neighborhood: ______________
01 First [urban] neighborhood
02 Second [urban] neighborhood
03 Third suburban village (neighborhood)
04 Fourth suburban village (neighborhood)
05 Fifth [urban] neighborhood
06 Sixth [urban] neighborhood
07 Seventh suburban village (neighborhood)
08 Eighth suburban village (neighborhood)
09 Ninth [urban] neighborhood
10 Tenth [urban] neighborhood
11 Eleventh suburban village (neighborhood)
12 Twelfth suburban village (neighborhood)

Village (Town): ____________ ☐☐T4
01 County town neighborhood
02 First village
03 Second village
04 Third village
05 County town neighborhood
06 Fourth village
07 Fifth village
08 Sixth village
09 County town neighborhood
10 Seventh village
11 Eighth village
12 Ninth village

Name of Respondent: ____________

Interview Date: ____Year _____Month ____Day ☐☐☐☐☐☐☐☐T7

Completion Evaluation: 1 Good 2 OK 3 Poor ☐CO

Interviewer Name: ________ Number: __________ ☐☐T6c

Supervisor Name: _________ Number: __________ ☐☐T6d

One Community questionnaire should be completed for each village/neighborhood. Note that each community (village or neighborhood) is a government-designated administrative district, not a natural population cluster. The Community questionnaire includes the following sections:

INFRASTRUCTURE, SERVICES AND ORGANIZATION


| | | |
|---|---|---|
| I | Community Background (ask community head) | 2 |
| II | Demographics (ask community head and community accountant) | 3 |
| III | TV Channels (ask community head) | 5 |
| IV | Child Care Facilities and Schools (ask community head) | 7 |
| V | Large Stores, Supermarkets, Hypermarkets and Cooperatives: Presence, Location, Size and Impact on Other Food Retailers (ask community head) | 8 |
| VI | Free Markets: Presence, Location and Size (ask community head) | 11 |
| VII | Supermarket/Hypermarket to which Most Residents in this Village/Neighborhood Go To Shop (ask Center for Disease Control food safety/inspection staff) | 12 |
| VIII | Fast Food Restaurants (ask community head) | 13 |
| IX | Recreational Facilities (ask community head) | 13 |
| X | Other Facilities and Services (ask community head) | 14 |
| XI | Medical Insurance (ask community health worker) | 16 |
| XII | Health Facilities (ask community health worker) | 17 |
| XIII | Family Planning (ask community family planning head) | 20 |


PRICES OF FOOD AND SPECIFIC LIVING MATERIAL
(ask community head or appropriate vendor/salesperson)


| | | |
|---|---|---|
| I | Food Grains | 22 |
| II | Cooking Oil and Sugar | 22 |
| III | Vegetables and Fruits | 23 |
| IV | Meat and Poultry | 23 |
| V | Fresh Milk | 23 |
| VI | Preserved Milk Products | 23 |
| VII | Fish | 24 |
| VIII | Bean Curd | 24 |
| IX | Fuel | 24 |
| X | Other Products | 25 |

**INFRASTRUCTURE, SERVICES AND ORGANIZATION**

**I. COMMUNITY BACKGROUND** (ask community head)

*** From the official records, obtain the following statistics:**

1. To which administrative district did this community belong in 2000? ☐O1a
    1 city neighborhood
    2 suburban neighborhood
    3 town neighborhood
    4 rural village

2. Has the administrative district of this community changed since 2000? ☐O1b
    0 no (skip to Question 5)
    1 yes
    9 unknown (skip to Question 5)

3. What is the current administrative district of this community? ☐O1c
    1 city neighborhood
    2 suburban neighborhood
    3 town neighborhood
    4 rural village
    5 other (specify: ___________)

4. In which year was the administrative district of the community changed? ☐☐☐☐O1d

5. Has the geographic boundary of this community changed since 1989? ☐O256
    0 no (skip to Table 1)
    1 yes

6. In which year was the geographic boundary changed? ☐☐☐☐O257

*** Ask Questions 8-9 for each year and record the answers in Table 1.**

*** Please answer these questions about your village/neighborhood, not your county/city.**

**Table 1. Population and Area Size**

| 7<br>Year | 8<br>What was the population of the village/neighborhood in this year?<br>* If "unknown," record -9999. | 9<br>What was the area of the village/ neighborhood in this year? (square km)<br>* If "unknown," record -99.99. |
|---|---|---|
| 1989 | **O258** ☐☐☐☐☐ | **O259** ☐☐☐.☐☐ |
| 1991 | **O260** ☐☐☐☐☐ | **O261** ☐☐☐.☐☐ |
| 1993 | **O262** ☐☐☐☐☐ | **O263** ☐☐☐.☐☐ |
| 1997 | **O264** ☐☐☐☐☐ | **O265** ☐☐☐.☐☐ |
| 2000 | **O266** ☐☐☐☐☐ | **O267** ☐☐☐.☐☐ |
| 2004 | **O268** ☐☐☐☐☐ | **O269** ☐☐☐.☐☐ |

10. How many households are in this village/neighborhood? ☐☐☐☐O0a

11. What is the total population of this county/city? ☐☐☐☐☐☐☐☐O270
* If the community is a town neighborhood/rural village, record the total population of the county. If the community is an urban/suburban neighborhood, record the total population of the city.

12. What is the total area of this county/city? (square km) ☐☐☐☐.☐☐O271
* If the community is a town neighborhood/rural village, record the total area of the county. If the community is an urban/suburban neighborhood, record the total area of the city.

*** Ask Questions 13-15 for rural villages/suburban neighborhoods only.**

13. Is this village/neighborhood located in a model township? ☐O9k
    0 no (skip to the next section)
    1 yes

14. At what level is this town a model? ☐O9l
    1 county
    2 provincial
    3 national
    4 other (specify: ___________)

15. What type of model is it? (there may be more than one type)

| | | | | | |
|---|---|---|---|---|---|
| (1) Economic development | 0 no | 1 yes | 9 unknown | ☐O9m_1 |
| (2) Environment and sanitation | 0 no | 1 yes | 9 unknown | ☐O9m_2 |
| (3) Family planning | 0 no | 1 yes | 9 unknown | ☐O9m_3 |
| (4) Administrative | 0 no | 1 yes | 9 unknown | ☐O9m_4 |
| (5) Other (specify: ___________) | 0 no | 1 yes | 9 unknown | ☐O9m_5 |

**II. DEMOGRAPHICS** (ask community head and community accountant)
*** Ask Question 1 for rural villages/suburban neighborhoods only.**

1. What was the average income (yuan) per person in this village/neighborhood in:
    (1) 2001 ___________ yuan ☐☐☐☐☐O9n
    (2) 2002 ___________ yuan ☐☐☐☐☐O9o
    (3) 2003 ___________ yuan ☐☐☐☐☐O9p

*** Ask Questions 3-4 about each occupation and record the answers in Table 2.**

**Table 2. Common Occupations**

| 2<br>Occupation | 3<br>What is the daily wage for this occupation in the village/neighborhood? (yuan)<br>* If "unknown," record -99. | 4<br>In addition to this wage, is free food provided?<br>0 no 1 yes 9 unknown |
|---|---|---|
| Ordinary male worker | **O47a** ☐☐☐ | **O47b** ☐ |
| Ordinary female worker | **O47c** ☐☐☐ | **O47d** ☐ |
| Primary school teacher | **O272** ☐☐☐ | **O273** ☐ |
| Middle school teacher | **O274** ☐☐☐ | **O275** ☐ |
| Nanny hired by households | **O48** ☐☐☐ | **O48a** ☐ |
| Ordinary construction worker | **O49** ☐☐☐ | **O50** ☐ |

5. What is the monthly income for a driver who is employed by a work unit and lives in this village/neighborhood? (yuan) ☐☐☐☐O51

6. Is there an open trade area, an open city, or a special economic zone near this village/neighborhood (within two hours by bus)? ☐O40
   0 no
   1 yes

7. What percentage of the work force in this village/neighborhood:
   (1) Is engaged mainly in agricultural activity (%) ☐☐☐O42
   (2) Worked out of town for > 1 month last year (%) ☐☐☐O43
   (3) Works in enterprises employing ≥ 20 people (%) ☐☐☐O44
   (4) Works in enterprises employing < 20 people (%) ☐☐☐O45

8. Are there any enterprises run by the village/neighborhood? ☐O52
   0 no (skip to Question 16)
   1 yes

9. How many such enterprises are there? ☐☐☐O53

10. What percentage of enterprises in this village/neighborhood are run by the village/neighborhood? (%) ☐☐☐O63

*** Ask Questions 13-15 for rural villages/suburban neighborhoods only.**

*** Ask Questions 13-14 about each subsidy and record the answers in Table 3.**

**Table 3. Subsidies Supported by Village/Neighborhood Enterprises**

| 11<br>Item Number | 12<br>Program | 13<br>Do revenues collected from village/neighborhood enterprises fund this welfare benefit/subsidy or service?<br>0 no 1 yes<br>* If "no," skip down to next item. | 14<br>What percentage of the total revenue is devoted to this service? (%) |
|---|---|---|---|
| 1 | Housing subsidy | **O64**<br>☐ | **O65**<br>☐☐.☐☐ |
| 2 | Health insurance | ☐ | ☐☐.☐☐ |
| 3 | Education subsidies | ☐ | ☐☐.☐☐ |
| 4 | Irrigation | ☐ | ☐☐.☐☐ |
| 5 | Road repair/construction | ☐ | ☐☐.☐☐ |
| 6 | Farm inputs and equipment renovation, maintenance, etc. | ☐ | ☐☐.☐☐ |
| 7 | Pensions, retirement salary, death compensation | ☐ | ☐☐.☐☐ |
| 8 | Grain subsidy | ☐ | ☐☐.☐☐ |
| 9 | Electricity subsidy | ☐ | ☐☐.☐☐ |

15. How many households in this village/neighborhood are specialized households? ☐☐☐O54

16. How many self-employed household enterprises are in this village/neighborhood? ☐☐☐O56

17. How many private enterprises are in this village/neighborhood? ☐☐☐O56a

18. Is there farm land in this village/neighborhood? ☐O57
    0 no (skip to Question 20)
    1 yes

19. What percentage of the farm land is:
    (1) Irrigated (%) ☐☐☐O57a
    (2) Irrigated by unified irrigation (%) ☐☐☐O57b
    (3) Collectively plowed (%) ☐☐☐O57c

20. How many of these businesses are currently operating in this village/neighborhood?
    (1) Fast food restaurants (e.g., McDonald's, Kentucky Fried Chicken) ☐☐O276
    (2) Other indoor restaurants ☐☐O60
    (3) Outdoor fixed food stalls ☐☐O61
    (4) Mobile food carts that sell cooked food (e.g., dumplings, steamed bread, pancakes) ☐☐O62
    (5) Bakeries and vendors that sell fried, twisted bread or other breakfast foods ☐☐O277
    (6) Bars and vendors that sell alcoholic beverages ☐☐O278
    (7) Ice cream parlors ☐☐O279
    (8) Fruit/vegetable stores and vendors (excluding seasonal vendors) ☐☐O280
    (9) Cafes (coffee or tea houses) ☐☐O281
    (10) Internet cafes ☐☐O282

**III. TV CHANNELS** (ask community head)

*** Ask Questions 3-4 about each TV channel and record the answers in Table 4.**

**Table 4. TV Channels**

| 1<br>Station Type | 2<br>Channel Name | 3<br>Is this channel available to residents in this village/neighborhood?<br>0 no 1 yes<br>* If "no," skip down to next item. | 4<br>In which year was this channel first available?<br>*If "unknown," record -999. |
|---|---|---|---|
| 1 Central TV | 101 News/Public Service Channel | **O92** | **O93** |
| | 102 Finance Channel | ☐ | ☐☐☐☐ |
| | 103 Arts Channel | ☐ | ☐☐☐☐ |
| | 104 International Channel | ☐ | ☐☐☐☐ |
| | 105 Sports Channel | ☐ | ☐☐☐☐ |
| | 106 Movie Channel | ☐ | ☐☐☐☐ |

| | | | |
|---|---|---|---|
| | 107 Military/Agriculture/Children's Channel | □ | □□□□ |
| | 108 TV Series and TV Movie Channel | □ | □□□□ |
| | 109 Educational Channel | □ | □□□□ |
| | 110 English Language Channel | □ | □□□□ |
| | 111 Science Channel | □ | □□□□ |
| | 112 Traditional Chinese Opera and Music Channel | □ | □□□□ |
| | 113 Western China Channel | □ | □□□□ |
| | 114 Children's Channel | □ | □□□□ |
| | 115 Popular Music Channel | □ | □□□□ |
| | 116 News Channel | □ | □□□□ |
| 2 Local TV | 201 Province | □ | □□□□ |
| | 202 City | □ | □□□□ |
| | 203 County | □ | □□□□ |
| 3 Satellite TV | 301 Beijing | □ | □□□□ |
| | 302 Tianjin | □ | □□□□ |
| | 303 Hebei | □ | □□□□ |
| | 304 Shanxi | □ | □□□□ |
| | 305 Inner Mongolia | □ | □□□□ |
| | 306 Liaoning | □ | □□□□ |
| | 307 Jilin | □ | □□□□ |
| | 308 Heilongjiang | □ | □□□□ |
| | 309 Shanghai | □ | □□□□ |
| | 310 Jiangsu | □ | □□□□ |
| | 311 Zhejiang | □ | □□□□ |
| | 312 Anhui | □ | □□□□ |
| | 313 Fujian | □ | □□□□ |
| | 314 Jiangxi | □ | □□□□ |
| | 315 Shandong | □ | □□□□ |
| | 316 Henan | □ | □□□□ |
| | 317 Hubei | □ | □□□□ |

| | | | |
|---|---|---|---|
| | 318 Hunan | ☐ | ☐☐☐☐ |
| | 319 Guangdong | ☐ | ☐☐☐☐ |
| | 320 Guangxi | ☐ | ☐☐☐☐ |
| | 321 Hainan | ☐ | ☐☐☐☐ |
| | 322 Sichuan | ☐ | ☐☐☐☐ |
| | 323 Chongqing | ☐ | ☐☐☐☐ |
| | 324 Guizhou | ☐ | ☐☐☐☐ |
| | 325 Yunnan | ☐ | ☐☐☐☐ |
| | 326 Tibet | ☐ | ☐☐☐☐ |
| | 327 Shaanxi | ☐ | ☐☐☐☐ |
| | 328 Gansu | ☐ | ☐☐☐☐ |
| | 329 Qinghai | ☐ | ☐☐☐☐ |
| | 330 Ningxia | ☐ | ☐☐☐☐ |
| | 331 Xinjiang | ☐ | ☐☐☐☐ |
| | 332 Hong Kong | ☐ | ☐☐☐☐ |
| | 334 China Entertainment TV | ☐ | ☐☐☐☐ |
| | 333 Other (specify: _______) | ☐ | ☐☐☐☐ |
| 4 Cable TV | 400 Cable TV | ☐ | ☐☐☐☐ |

## IV. CHILD CARE FACILITIES AND SCHOOLS (ask community head)

*** Ask Questions 2-4 about each type of facility and record the answers in Table 5.**

**Table 5. Child Care Facilities**

| 1<br>Age Group | 2<br>Is there a child care facility for this age group in this village/neighborhood?<br>0 no 1 yes<br>* If "yes," skip to Question 4. | 3<br>If "no," how far away is the nearest child care facility of this type? (km) | 4<br>What is the average monthly fee for this age group? (yuan) |
|---|---|---|---|
| < 3 years | **O67a**<br>☐ | **O68a**<br>☐☐.☐ | **O69a**<br>☐☐☐.☐ |
| 3-6 years | ☐ | ☐☐.☐ | ☐☐☐.☐ |

***Ask Questions 5-8 for rural villages/suburban neighborhoods only.**

5. Is there a preschool for children managed by a primary school in this village/neighborhood? ☐O76
   0 no (skip to Table 6)
   1 yes

6. What is the minimum age of children who can go to this school? (years) ☐O76a

7. Is there a charge for a child to attend this preschool? ☐O77
   0 no (skip to Table 6)
   1 yes

8. What is the fee per child per semester, excluding food? (yuan) ☐☐☐☐☐O78a

*** Ask Questions 10-11 about each type of school and record the answers in Table 6**.

**Table 6. Public Schools**

| 9<br>School Type | 10<br>Is there a school of this type in this village/neighborhood?<br>0 no 1 yes 9 unknown<br>* If "yes," skip down to next item. | 11<br>If "no," how far away is the nearest school of this type? (km)<br>* If "unknown," record -99.9. |
|---|---|---|
| Primary school | **O79** ☐ | **O80** ☐☐☐.☐ |
| Lower middle school | **O81** ☐ | **O82** ☐☐☐.☐ |
| Upper middle school | **O83** ☐ | **O84** ☐☐☐.☐ |
| Vocational upper middle school or vocational technical school | **O85** ☐ | **O86** ☐☐☐.☐ |

## V. LARGE STORES, SUPERMARKETS, HYPERMARKETS AND COOPERATIVES: PRESENCE, LOCATION, SIZE AND IMPACT ON OTHER FOOD RETAILERS

(ask community head)
* A "hypermarket" is a very large store that sells products found in supermarkets as well as products commonly found in department stores.

*** Ask Questions 3-6 about each product and record the answers in Table 7.**

3. To what large store/supermarket/hypermarket/cooperative do residents in this village/neighborhood go most often to buy this product?
   * Record the store name in item 3 in Table 7.

4. What type of store is this?
   1 Western-owned supermarket/hypermarket (e.g., Metro, Makro, Carrefour, Wal-Mart, Auchan)
   2 Chinese-owned supermarket/hypermarket (e.g., Lianhua, Hualian, Huarun-Wanjia, Wumei)
   3 other Asian-owned supermarket/hypermarket (e.g., Park 'n Shop, Trust-Mart, Lotus, Acon/Jusco, Ito-Yokado)
   4 other large store or cooperative store
   5 other store
   9 never buy or no store available

5. Where is this large store/supermarket/hypermarket/cooperative?
   1 in this village/neighborhood
   2 in this city but a different neighborhood
   3 in another village/town/city
   9 never buy or no store available

6. How far away is this large store/supermarket/hypermarket/cooperative? (km)
   * If in this village/neighborhood, record 00.0. Otherwise, record the actual distance.

**Table 7. Large Stores/Supermarkets/Hypermarkets/Cooperatives Where You Buy Food and Other Products**

| 1<br>Item<br>Number | 2<br>Item Name | 3<br>Store Name | 4<br>Type | 5<br>Location | 6<br>Distance<br>(km) |
|---|---|---|---|---|---|
| 1 | Food grains | | **O283**<br>☐ | **O13a**<br>☐ | **O14a**<br>☐☐.☐ |
| 2 | Cooking oil | | ☐ | ☐ | ☐☐.☐ |
| 3 | Vegetables | | ☐ | ☐ | ☐☐.☐ |
| 3a | Fruits | | ☐ | ☐ | ☐☐.☐ |
| 4 | Meat, poultry and eggs | | ☐ | ☐ | ☐☐.☐ |
| 5 | Fresh milk | | ☐ | ☐ | ☐☐.☐ |
| 6 | Preserved milk (canned milk, infant formula, etc.) | | ☐ | ☐ | ☐☐.☐ |
| 7 | Fish | | ☐ | ☐ | ☐☐.☐ |
| 8 | Bean curd | | ☐ | ☐ | ☐☐.☐ |
| 10 | Fuel (coal, gas, natural gas, etc.) | | ☐ | ☐ | ☐☐.☐ |
| 11 | Cigarettes | | ☐ | ☐ | ☐☐.☐ |
| 12 | Alcohol | | ☐ | ☐ | ☐☐.☐ |
| 13 | Soft drinks | | ☐ | ☐ | ☐☐.☐ |

7. How many supermarkets or hypermarkets are within a 30-minute bus ride from this village/neighborhood? ☐☐O284

8. Where is the supermarket/hypermarket to which most residents in this village/neighborhood go to shop? ☐O285
   1 in this village/neighborhood
   2 in this city but a different neighborhood
   3 in another village/town/city
   9 no supermarket/hypermarket available (skip to Question 11)

9. How far away is this supermarket/hypermarket? (km) ☐☐.☐O286
   * If in this village/neighborhood, record 00.0. Otherwise, record the actual distance.

10. Is this supermarket/hypermarket Western-owned, Chinese-owned, or owned by another Asian country (e.g., Thailand, Malaysia, Japan)? ☐O287
    1 Western-owned (e.g., Metro, Makro, Carrefour, Wal-Mart, Auchan)
    2 Chinese-owned (e.g., Lianhua, Hualian, Huarun-Wanjia, Wumei)
    3 other-Asian-owned (e.g., Park ’n Shop, Trust-Mart, Lotus, Acon/Jusco, Ito-Yokado)
    9 unknown

11. Where is the Western-owned supermarket/hypermarket nearest this village/neighborhood? ☐O288
   1 in this village/neighborhood
   2 in this city but a different neighborhood
   3 in another village/town/city
   9 no Western supermarket/hypermarket available (skip to Question 13)

12. How far away is this Western supermarket/hypermarket? (km) ☐☐.☐O289
   * If in this village/neighborhood, record 00.0. Otherwise, record the actual distance.

13. Where is the Chinese-owned supermarket/hypermarket nearest this village/neighborhood? ☐O290
   1 in this village/neighborhood
   2 in this city but a different neighborhood
   3 in another village/town/city
   9 no Chinese supermarket/hypermarket available (skip to Question 15)

14. How far away is this Chinese supermarket/hypermarket? (km) ☐☐.☐O291
   * If in this village/neighborhood, record 00.0. Otherwise, record the actual distance.

15. Where is the other-Asian-owned supermarket/hypermarket (Thai, Malaysian, Japanese) near this village/neighborhood? ☐O292
   1 in this village/neighborhood
   2 in this city but a different neighborhood
   3 in another village/town/city
   9 no other-Asian supermarket/hypermarket available (skip to Question 17)

16. How far away is this other-Asian supermarket/hypermarket? (km) ☐☐.☐O293
   * If in this village/neighborhood, record 00.0. Otherwise, record the actual distance.

17. During the past 3 years, how many food retailers within a 30-minute bus ride have gone out of business? ☐☐O294

18. During the past 3 years, how many new supermarkets/hypermarkets within a 30-minute bus ride have opened? ☐☐O295

*** Ask Questions 20-21 about each product and record the answers in Table 8.**

**Table 8. Nontraditional Foods Commonly Found in Supermarkets/Hypermarkets**

| 19<br>Food item | 20<br>Is this food commonly found in supermarkets/hypermarkets?<br>0 no 1 yes<br>9 no supermarket/hypermarket available | 21<br>Is this food commonly found in free markets?<br>0 no 1 yes<br>9 no free market available |
|---|---|---|
| Non-sweetened instant cereals | **O296** ☐ | **O297** ☐ |
| Sweetened instant cereals | **O298** ☐ | **O299** ☐ |
| Potato chips or sticks | **O300** ☐ | **O301** ☐ |
| Chocolate or chocolate snack bars | **O302** ☐ | **O303** ☐ |
| Healthy snack foods (low in fat or sugar, according to label) | **O304** ☐ | **O305** ☐ |
| Cow's milk (fresh) | **O306** ☐ | **O307** ☐ |

**VI. FREE MARKETS: PRESENCE, LOCATION AND SIZE** (ask community head)

*** Ask Questions 3-7 about each product and record the answers in Table 9.**

3. To what free market do residents in this village/neighborhood go most often to buy this product?
   * Record the market name in item 3 in Table 9. If the free market has no name, record its accurate address.

4. Where is this free market?
   1 in this village/neighborhood
   2 in this city but a different neighborhood
   3 in another village/town/city
   9 never buy or no market available

5. How far away is this free market? (km)
   * If in this village/neighborhood, record 00.0. Otherwise, record the actual distance.

6. How many days per week is this free market open?

7. Do residents in this village/neighborhood purchase this item more often in this free market than in supermarkets/hypermarkets?
   0 no
   1 yes

**Table 9. Free Markets Where You Buy Food and Other Products**

| 1<br>Item Number | 2<br>Item Name | 3<br>Free Market Name | 4<br>Location | 5<br>Distance (km) | 6<br>Days/ Week | 7<br>More Often Purchased Here? |
|---|---|---|---|---|---|---|
| 1 | Food grains | | **O16**<br>☐ | **O17**<br>☐☐.☐ | **O17a**<br>☐ | **O308**<br>☐ |
| 2 | Cooking oil | | ☐ | ☐☐.☐ | ☐ | ☐ |
| 3 | Vegetables | | ☐ | ☐☐.☐ | ☐ | ☐ |
| 3a | Fruits | | ☐ | ☐☐.☐ | ☐ | ☐ |
| 4 | Meat, poultry and eggs | | ☐ | ☐☐.☐ | ☐ | ☐ |
| 5 | Fresh milk | | ☐ | ☐☐.☐ | ☐ | ☐ |
| 6 | Preserved milk (canned milk, infant formula, etc.) | | ☐ | ☐☐.☐ | ☐ | ☐ |
| 7 | Fish | | ☐ | ☐☐.☐ | ☐ | ☐ |
| 8 | Bean curd | | ☐ | ☐☐.☐ | ☐ | ☐ |
| 10 | Fuel (coal, gas, natural gas, etc.) | | ☐ | ☐☐.☐ | ☐ | ☐ |
| 11 | Cigarettes | | ☐ | ☐☐.☐ | ☐ | ☐ |
| 12 | Alcohol | | ☐ | ☐☐.☐ | ☐ | ☐ |
| 13 | Soft drinks | | ☐ | ☐☐.☐ | ☐ | ☐ |

8. How many free markets are within a 30-minute bus ride from this village/neighborhood? ☐☐O309

9. Where is the free market nearest this village/neighborhood? ☐O310
   1 in this village/neighborhood
   2 in this city but a different neighborhood
   3 in another village/town/city
   9 no free market available (skip to Question 12)

10. How far away is this free market? (km) ☐☐.☐O311
    * If in this village/neighborhood, record 00.0. Otherwise, record the actual distance.

11. Is this free market open-air or enclosed? ☐O312
    1 open-air
    2 enclosed

12. How many vendors are currently operating in the largest free market to which residents in this village/neighborhood go to shop? ☐☐☐O313

13. If the following foods are available from supermarkets/hypermarkets, are residents in this village/neighborhood less likely to purchase them from free markets?

| | | | | | | |
|---|---|---|---|---|---|---|
| (1) | Food grains | 0 | no | 1 | yes | ☐O314 |
| (2) | Fruits | 0 | no | 1 | yes | ☐O315 |
| (3) | Vegetables | 0 | no | 1 | yes | ☐O316 |
| (4) | Snack foods | 0 | no | 1 | yes | ☐O317 |
| (5) | Packaged[/instant] foods | 0 | no | 1 | yes | ☐O318 |

*** Check Section V/Question 8 on page 9.**
**If Q8=1,2,3: [Visit this supermarket/hypermarket and] fill in all information in Section VII below.**
**If Q8=9: Skip to Section VIII.**

## VII. SUPERMARKET/HYPERMARKET TO WHICH MOST RESIDENTS IN THIS VILLAGE/NEIGHBORHOOD GO TO SHOP

(ask Center for Disease Control food safety/inspection staff)

1. How big is this supermarket/hypermarket?
   (1) Total number of floors ☐☐O319
   (2) Total area of all floors (square meters) ☐☐☐☐O320

*** Ask Questions 3-4 about each product and record the answers in Table 10.**

**Table 10. Supermarket/Hypermarket Food Retail Environment: Variety and Availability**

| 2<br>Food item | 3<br>How many different types of this food are available in this supermarket/hypermarket? | 4<br>How much total shelf space is used for this food (square meters)? |
|---|---|---|
| Fresh fruit | **O321** ☐☐☐ | **O322** ☐☐ |
| Fresh vegetables | **O323** ☐☐☐ | **O324** ☐☐ |
| Snack foods, candy | **O325** ☐☐☐ | **O326** ☐☐ |

**VIII. FAST FOOD RESTAURANTS** (ask community head)

1. Are there any fast food restaurants, such as McDonald's or Kentucky Fried Chicken, near this village/neighborhood? ☐O327
   - 0 no (skip to the next section)
   - 1 yes
   - 9 unknown (skip to the next section)

*** Ask Questions 3-5 about each restaurant and record the answers in Table 11.**

**Table 11. Fast Food Restaurants**

| 2<br>Item Number | 3<br>What is the name of this fast food restaurant? | 4<br>Where is this restaurant?<br>1 in this village/neighborhood<br>2 in this city but a different neighborhood<br>3 in another village/town/city<br>9 unknown or no restaurant of this type available<br>* If code 9 is used, skip down to next item. | 5<br>How far away is this restaurant? (km)<br>* If in this village/neighborhood, record 000.0. Otherwise, record the actual distance. |
|---|---|---|---|
| 1 | McDonald's | **O248**<br>☐ | **O249**<br>☐☐☐.☐ |
| 2 | Kentucky Fried Chicken | ☐ | ☐☐☐.☐ |
| 2a | Pizza Hut | ☐ | ☐☐☐.☐ |
| 3 | Other (specify: _______ ) | ☐ | ☐☐☐.☐ |
| 4 | Other (specify: _______ ) | ☐ | ☐☐☐.☐ |

**IX. RECREATIONAL FACILITIES** (ask community head)

1. Where is the gym or exercise center nearest this village/neighborhood? ☐O250
   - 1 in this village/neighborhood
   - 2 in this city but a different neighborhood
   - 3 in another village/town/city
   - 9 no gym/exercise center available (skip to Question 3)

2. How far away is this gym/exercise center? (km) ☐☐.☐O251
   * If in this village/neighborhood, record 00.0. Otherwise, record the actual distance.

3. Where is the park/public recreation place nearest this village/neighborhood? ☐O252
   * Parks/public recreation places are spaces with or without facilities for children or adults to play or participate in sports/physical activities.
   - 1 in this village/neighborhood
   - 2 in this city but a different neighborhood
   - 3 in another village/town/city
   - 9 no park/public recreation place available (skip to Question 6)

4. How far away is this park/public recreation place? (km) ☐☐.☐O253
   * If in this village/neighborhood, record 00.0. Otherwise, record the actual distance.

5. Does this park/public recreation place have the following facilities?

| | | | | | |
|---|---|---|---|---|---|
| (1) | Soccer fields | 0 no | 1 yes | 9 unknown | ☐O328 |
| (2) | Basketball courts | 0 no | 1 yes | 9 unknown | ☐O329 |
| (3) | Volleyball, badminton or tennis courts | 0 no | 1 yes | 9 unknown | ☐O330 |
| (4) | Running or walking trails | 0 no | 1 yes | 9 unknown | ☐O331 |
| (5) | Ping pong tables | 0 no | 1 yes | 9 unknown | ☐O332 |
| (6) | Other special sports facilities (specify:____) | 0 no | 1 yes | 9 unknown | ☐O333 |

6. Where is the playground nearest this village/neighborhood which is accessible to most residents? ☐O334
* Playgrounds are spaces with facilities for children or adults to play or participate in sports/physical activities which are maintained by an institution, school, or government department but may have restricted use (e.g., employees of the institution or students of the school only).
   1 in this village/neighborhood
   2 in this city but a different neighborhood
   3 in another village/town/city
   9 no playground available (skip to the next section)

7. How far away is this playground? (km) ☐☐.☐O335
* If in this village/neighborhood, record 00.0. Otherwise, record the actual distance.

8. Does this playground have the following facilities?

| | | | | | |
|---|---|---|---|---|---|
| (1) | Soccer fields | 0 no | 1 yes | 9 unknown | ☐O336 |
| (2) | Basketball courts | 0 no | 1 yes | 9 unknown | ☐O337 |
| (3) | Volleyball, badminton or tennis courts | 0 no | 1 yes | 9 unknown | ☐O338 |
| (4) | Running or walking trails | 0 no | 1 yes | 9 unknown | ☐O339 |
| (5) | Ping pong tables | 0 no | 1 yes | 9 unknown | ☐O340 |
| (6) | Other special sports facilities (specify:_____) | 0 no | 1 yes | 9 unknown | ☐O341 |

**X. OTHER FACILITIES AND SERVICES** (ask community head)

1. Are there fewer residents who bike to work now than in 2000 in this village/neighborhood? ☐O254
   0 no (skip to Question 3)
   1 yes

2. Why did they stop biking to work?

| | | | | | |
|---|---|---|---|---|---|
| (1) | Environmental pollution | 0 no | 1 yes | 9 unknown | ☐O255_1 |
| (2) | Too much traffic or traffic too dangerous | 0 no | 1 yes | 9 unknown | ☐O255_2 |
| (3) | Fewer bicycle lanes now | 0 no | 1 yes | 9 unknown | ☐O255_3 |
| (3a) | Drive car/take taxi now | 0 no | 1 yes | 9 unknown | ☐O255_3a |
| (4) | Other (specify: __________) | 0 no | 1 yes | 9 unknown | ☐O255_4 |

3. What is the most common characteristic of the roads in or around this village/neighborhood? ☐O23
   1 dirt
   2 stone, gravel, or mixed material (skip to Question 6)
   3 paved road (skip to Question 6)

4. Are there any stone, gravel, or paved roads? ☐O23a
   0 no
   1 yes (skip to Question 6)

5. How far away is the nearest stone, gravel, or paved road? (km) ☐☐.☐O24

6. Are the following services available in this village/neighborhood?

| | | | | | |
|---|---|---|---|---|---|
| (1) | Convenient telegraph service | 0 no | 1 yes | 9 unknown | ☐O25 |
| (2) | Convenient telephone service | 0 no | 1 yes | 9 unknown | ☐O26 |
| (3) | Cell phone service | 0 no | 1 yes | 9 unknown | ☐O342 |
| (4) | Convenient internet service | 0 no | 1 yes | 9 unknown | ☐O343 |
| (5) | Convenient fax service | 0 no | 1 yes | 9 unknown | ☐O26a |
| (6) | Postal service | 0 no | 1 yes | 9 unknown | ☐O27 |
| (7) | Provincial daily newspaper received on the day it is published | 0 no | 1 yes | 9 unknown | ☐O28 |
| (8) | Convenient movies | 0 no | 1 yes | 9 unknown | ☐O29 |

7. Is electricity available in this village/neighborhood? ☐O30
   0 no (skip to Question 10)
   1 yes

8. When electricity is supplied, how many hours per day, on the average, is electricity available? (hours) ☐☐O31

9. On the average, how many days per week is the electricity cut off? ☐O32

10. Is there a bus stop (or long distance bus stop) in this village/neighborhood? ☐O33
    0 no
    1 yes (skip to Question 13)

11. How far away is the nearest bus stop? (km) ☐☐.☐O34

12. How often does the bus serve this bus stop? (minutes) ☐☐☐O344

13. Is this village/neighborhood near a train station? ☐O35
    0 no
    1 yes (skip to Question 15)

14. How far away is the nearest train station? (km) ☐☐☐.☐O36

15. Are public baths available in this village/neighborhood? ☐O37
    0 no
    1 yes (skip to Question 17)

16. How far away is the nearest public bath? (km) ☐☐.☐O38

17. Is this village/neighborhood near a navigable river? ☐O39
    0 no
    1 yes

**XI. MEDICAL INSURANCE** (ask community health worker)

*** Ask Questions 3-4 about each type of medical insurance and record the answers in Table 12.**

**Table 12. Medical Insurance**

| 1<br>Item Number | 2<br>Insurance Type | 3<br>Does this village/ neighborhood have this type of medical insurance?<br>0 no 1 yes 9 unknown<br>* If "no" or "unknown," skip down to next item. | 4<br>In which year was this insurance first available?<br>* If "unknown," record -999. |
|---|---|---|---|
| | | **O8cb** | **O8cc** |
| 0 | Commercial insurance | ☐ | ☐☐☐☐ |
| 1 | Free medical service | ☐ | ☐☐☐☐ |
| 2 | Worker's compensation | ☐ | ☐☐☐☐ |
| 3 | Insurance for family members | ☐ | ☐☐☐☐ |
| 4 | Cooperative insurance | ☐ | ☐☐☐☐ |
| 5 | Unified planning medical service | ☐ | ☐☐☐☐ |
| 6 | Health insurance for women and children | ☐ | ☐☐☐☐ |
| 7 | EPI (expanded program of immunization) insurance for children | ☐ | ☐☐☐☐ |
| 8 | Other (specify: _________) | ☐ | ☐☐☐☐ |

**XII. HEALTH FACILITIES** (ask community health worker)

*** Ask Questions 2-9 about each health facility and record the answers in Table 13.**

2. When residents in this village/neighborhood need health services, what health facilities can they use?
   * Record the name of each facility in item 2 in Table 13.

3. What type of facility is this?

| | | | |
|---|---|---|---|
| 01 | village clinic | 09 | city maternal and child hospital |
| 02 | private clinic | 10 | city hospital |
| 03 | work unit clinic | 11 | worker's hospital |
| 04 | other clinic | 12 | other hospital |
| 05 | town family planning service | 13 | drug store |
| 06 | town hospital | 15 | other (specify: ___________) |
| 07 | county maternal and child hospital | - 9 | unknown |
| 08 | county hospital | | |

4. Where is this facility?
   1 in this village/neighborhood
   2 in this city but a different neighborhood
   3 in another village/town/city

5. How far away is this facility? (km)
   * If in this village/neighborhood, record 000.0. Otherwise, record the actual distance.

6. On the average, how many hours per week is this facility open for business?

7. How many doctors does this facility have?

8. How many hospital beds does this facility have?

9. How much is the registration fee at this facility? (yuan)

*** If respondent does not report at least 1 clinic, go back to Question 2 and ask about the most commonly used clinic. If respondent does not report at least 1 hospital, go back to Question 2 and ask about the most commonly used hospital.**

**Table 13. Health Facilities**

| 1 Facility Number | 2 Facility Name | 3 Facility Type | 4 Location | 5 Distance (km) | 6 Business Hours (hrs/wk) | 7 Number of Doctors | 8 Number of Hospital Beds | 9 Registration Fee (yuan) |
|---|---|---|---|---|---|---|---|---|
| 1 | | **O19b** □□ | **O20** □ | **O21** □□□.□ | **O21a** □□□ | **O21b** □□□□ | **O21c** □□□□ | **O21d** □□.□ |
| 2 | | □□ | □ | □□□.□ | □□□ | □□□□ | □□□□ | □□.□ |
| 3 | | □□ | □ | □□□.□ | □□□ | □□□□ | □□□□ | □□.□ |
| 4 | | □□ | □ | □□□.□ | □□□ | □□□□ | □□□□ | □□.□ |
| 5 | | □□ | □ | □□□.□ | □□□ | □□□□ | □□□□ | □□.□ |
| 6 | | □□ | □ | □□□.□ | □□□ | □□□□ | □□□□ | □□.□ |
| 7 | | □□ | □ | □□□.□ | □□□ | □□□□ | □□□□ | □□.□ |

10. Have any new clinics or hospitals opened in this village/neighborhood since 2000? ☐O21e
    0 no (skip to Question 19)
    1 yes

*** Ask Questions 12-18 about each new health facility and record the answers in Table 14.**

12. What is the name of this facility?
    * Record the name of each facility in item 12 of Table 14.

13. What type of facility is this?

| | | | |
|---|---|---|---|
| 01 | village clinic | 09 | city maternal and child hospital |
| 02 | private clinic | 10 | city hospital |
| 03 | work unit clinic | 11 | worker's hospital |
| 04 | other clinic | 12 | other hospital |
| 05 | town family planning service | 13 | drug store |
| 06 | town hospital | 15 | other (specify: ___________) |
| 07 | county maternal and child hospital | - 9 | unknown |
| 08 | county hospital | | |

14. Approximately when did this facility open for business? (year)

15. On the average, how many hours per week is this facility open for business?

16. How many doctors does this facility have?

17. How many hospital beds does this facility have?

18. How much is the registration fee at this facility? (yuan)

**Table 14. Newly Opened Health Facilities**

| 11<br>Facility Number | 12<br>Facility Name | 13<br>Facility Type | 14<br>Year Opened | 15<br>Business Hours (hrs/wk) | 16<br>Number of Doctors | 17<br>Number of Hospital Beds | 18<br>Registration Fee (yuan) |
|---|---|---|---|---|---|---|---|
| 1 | | **O231a**<br>☐☐ | **O232**<br>☐☐☐☐ | **O233**<br>☐☐☐ | **O234**<br>☐☐☐☐ | **O235**<br>☐☐☐☐ | **O236**<br>☐☐.☐ |
| 2 | | ☐☐ | ☐☐☐☐ | ☐☐☐ | ☐☐☐☐ | ☐☐☐☐ | ☐☐.☐ |
| 3 | | ☐☐ | ☐☐☐☐ | ☐☐☐ | ☐☐☐☐ | ☐☐☐☐ | ☐☐.☐ |
| 4 | | ☐☐ | ☐☐☐☐ | ☐☐☐ | ☐☐☐☐ | ☐☐☐☐ | ☐☐.☐ |

19. Did any clinic or hospital that residents from this village/neighborhood used regularly go out of business, or was any clinic/hospital replaced by another type of health facility since 2000? ☐O236a
    0 no (skip to the next section)
    1 yes

*** Ask Questions 21-27 about each health facility and record the answers in Table 15.**

21. What was the name of this facility?
    * Record the name of each facility in item 21 in Table 15.

22. What type of facility was this?

| | | | |
|---|---|---|---|
| 01 | village clinic | 09 | city maternal and child hospital |
| 02 | private clinic | 10 | city hospital |
| 03 | work unit clinic | 11 | worker's hospital |
| 04 | other clinic | 12 | other hospital |
| 05 | town family planning service | 13 | drug store |
| 06 | town hospital | 15 | other (specify: __________) |
| 07 | county maternal and child hospital | - 9 | unknown |
| 08 | county hospital | | |

23. Was this facility replaced by another type of facility?
    0 no (skip to Question 25)
    1 yes

24. Type of facility after replacement?

| | | | |
|---|---|---|---|
| 01 | village clinic | 09 | city maternal and child hospital |
| 02 | private clinic | 10 | city hospital |
| 03 | work unit clinic | 11 | worker's hospital |
| 04 | other clinic | 12 | other hospital |
| 05 | town family planning service | 13 | drug store |
| 06 | town hospital | 15 | other (specify: __________) |
| 07 | county maternal and child hospital | - 9 | unknown |
| 08 | county hospital | | |

25. Where was this facility?
    1 in this village/neighborhood
    2 in this city/town but a different neighborhood
    3 in another village/town/city

26. How far away was this facility? (km)
    * If in this village/neighborhood, record 000.0. Otherwise, record the actual distance.

27. In which year did this facility go out of business?

**Table 15. Health Facilities Closed or Replaced**

| 20 Facility Num-ber | 21 Facility Name | 22 Facility Type | 23 Replaced? | 24 Facility Type After Replacement | 25 Location | 26 Distance (km) | 27 Year Went Out of Business |
|---|---|---|---|---|---|---|---|
| 1 | | **O238a** □□ | **O239** □ | **O240a** □□ | **O241** □ | **O242** □□□.□ | **O243** □□□□ |
| 2 | | □□ | □ | □□ | □ | □□□.□ | □□□□ |
| 3 | | □□ | □ | □□ | □ | □□□.□ | □□□□ |

**XIII. FAMILY PLANNING** (ask community family planning head)

1. What percentage of the population in this village/neighborhood is from each of the following ethnic groups (nationalities)?
 * Percentages must add up to 100%.

| | | |
|---|---|---|
| (1) | Han (if 100%, [record 100, then] skip to Question 4) | ☐☐☐R6j_1 |
| (2) | Mongolian | ☐☐☐R6j_2 |
| (3) | Hui | ☐☐☐R6j_3 |
| (4) | Tibetan | ☐☐☐R6j_4 |
| (5) | Vaguer | ☐☐☐R6j_5 |
| (6) | Miao | ☐☐☐R6j_6 |
| (7) | Yi | ☐☐☐R6j_7 |
| (8) | Zhuang | ☐☐☐R6j_8 |
| (9) | Buyi | ☐☐☐R6j_9 |
| (10) | Korean | ☐☐☐R6j_10 |
| (11) | Man | ☐☐☐R6j_11 |
| (12) | Dong | ☐☐☐R6j_12 |
| (13) | Yao | ☐☐☐R6j_13 |
| (14) | Bai | ☐☐☐R6j_14 |
| (15) | Tujia | ☐☐☐R6j_15 |
| (16) | Hani | ☐☐☐R6j_16 |
| (17) | Hasake | ☐☐☐R6j_17 |
| (18) | Dai | ☐☐☐R6j_18 |
| (19) | Li | ☐☐☐R6j_19 |
| (20) | Other (specify: ___________) | ☐☐☐R6j_20 |

2. Is the family planning policy the same for minorities as it is for Han nationality? ☐R6b
 0 no
 1 yes

3. Are minority couples in this village/neighborhood allowed to have two children? ☐R6k
 0 no
 1 yes, but only if both the husband and wife are minorities
 2 yes, as long as either the husband or wife is a minority

4. Are all couples in this village/neighborhood allowed to have more than two children? ☐R6i
 0 no
 1 yes (skip to Question 7)

5. Are all couples in this village/neighborhood allowed to have two children? ☐R6
 0 no
 1 yes (skip to Question 7)

6. Are couples of Han nationality allowed to have two children if:

| | | | | | |
|---|---|---|---|---|---|
| (1) Their first child is a girl | 0 no | 1 yes | 9 unknown | ☐R1 |
| (2) Each parent is an only child | 0 no | 1 yes | 9 unknown | ☐R3 |
| (3) Both parents have certain special occupations | 0 no | 1 yes | 9 unknown | ☐R4 |
| (4) There are other exceptions (specify:_________) | 0 no | 1 yes | 9 unknown | ☐R5d |

7. Have the local cadres implemented the family planning responsibility system? ☐R20d

    0 no
    1 yes, connected with economic rewards
    2 yes, not connected with economic rewards

8. Do couples receive a subsidy if they have only one child? ☐R16

    0 no
    1 yes

9. Do one-child families receive child health care subsidies? ☐R33

    0 no
    1 yes

**PRICES OF FOOD AND SPECIFIC LIVING MATERIAL**
(ask community head or appropriate vendor/salesperson)

*** Obtain the following prices at the time of interview. If the village/neighborhood doesn't have the item or the price is "unknown," record -9.9.**

**Table I. Food Grains**

| Item Number | Food Name | Large Store Retail Price (yuan) | Free Market Price (yuan) |
|---|---|---|---|
| 1 | Good rice (per jin) | **P96** ☐☐.☐ | **P8** ☐☐.☐ |
| 2 | Rice, most commonly eaten (per jin) | ☐☐.☐ | ☐☐.☐ |
| 3 | Bleached flour (per jin) | ☐☐.☐ | ☐☐.☐ |
| 4 | Unbleached flour (per jin) | ☐☐.☐ | ☐☐.☐ |
| 5 | Noodles made of bleached flour (per jin) | ☐☐.☐ | ☐☐.☐ |
| 6 | Noodles made of unbleached flour (per jin) | ☐☐.☐ | ☐☐.☐ |
| 7 | Corn flour (per jin) | ☐☐.☐ | ☐☐.☐ |
| 8 | Millet (per jin) | ☐☐.☐ | ☐☐.☐ |
| 9 | Sorghum (per jin) | ☐☐.☐ | ☐☐.☐ |

**Table II. Cooking Oil and Sugar**

| Item Number | Food Name | Large Store Retail Price (yuan) | Free Market Price (yuan) |
|---|---|---|---|
| 1 | Rape seed oil (per jin) | **P97** ☐☐.☐ | **P16** ☐☐.☐ |
| 2 | Soybean oil (per jin) | ☐☐.☐ | ☐☐.☐ |
| 3 | Peanut oil (per jin) | ☐☐.☐ | ☐☐.☐ |
| 4 | Cottonseed oil (per jin) | ☐☐.☐ | ☐☐.☐ |
| 5 | Tea oil (per jin) | ☐☐.☐ | ☐☐.☐ |
| 6 | White sugar (per jin) | ☐☐.☐ | ☐☐.☐ |
| 7 | Eggs (per jin) | ☐☐.☐ | ☐☐.☐ |
| 8 | Soy sauce, most commonly used (per jin) | ☐☐.☐ | ☐☐.☐ |
| 9 | Vinegar, most commonly used (per jin) | ☐☐.☐ | ☐☐.☐ |
| 0 | Refined oil (per jin) | ☐☐.☐ | ☐☐.☐ |

**Table III. Vegetables and Fruits**

| Item Number | Food Name | Large Store Retail Price (yuan) | Free Market Price (yuan) |
|---|---|---|---|
| 1 | Green vegetables (rape) (per jin) | **P98** ☐☐.☐ | **P24** ☐☐.☐ |
| 2 | Cabbage (per jin) | ☐☐.☐ | ☐☐.☐ |
| 3 | Vegetable, most commonly eaten (per jin) (specify:__________) | ☐☐.☐ | ☐☐.☐ |
| 4 | Apple (per jin) | ☐☐.☐ | ☐☐.☐ |
| 5 | Orange (per jin) | ☐☐.☐ | ☐☐.☐ |

**Table IV. Meat and Poultry**

| Item Number | Food Name | Large Store Retail Price (yuan) | Free Market Price (yuan) |
|---|---|---|---|
| 1 | Pork, fatty and lean (per jin) | **P99** ☐☐.☐ | **P32** ☐☐.☐ |
| 2 | Pork, lean (per jin) | ☐☐.☐ | ☐☐.☐ |
| 3 | Live chicken (per jin) | ☐☐.☐ | ☐☐.☐ |
| 4 | Chicken, cleaned (per jin) | ☐☐.☐ | ☐☐.☐ |
| 5 | Beef (per jin) | ☐☐.☐ | ☐☐.☐ |
| 6 | Mutton (per jin) | ☐☐.☐ | ☐☐.☐ |

**Table V. Fresh Milk**

| Item Number | Food Name | Large Store Retail Price (yuan) | Free Market Price (yuan) |
|---|---|---|---|
| 1 | Fresh milk (**per package—250 ml**) | **P100** ☐☐.☐ | **P40** ☐☐.☐ |

**Table VI. Preserved Milk Products**

| Item Number | Food Name | Large Store Retail Price (yuan) | Free Market Price (yuan) |
|---|---|---|---|
| 1 | Condensed, sweetened (per jin) | **P101** ☐☐.☐ | **P48** ☐☐.☐ |
| 2 | Whole, powdered (per jin) | ☐☐.☐ | ☐☐.☐ |
| 3 | Substitute formula, soy or rice (per jin) | ☐☐.☐ | ☐☐.☐ |
| 4 | Infant formula (per jin) | ☐☐.☐ | ☐☐.☐ |

**Table VII. Fish**

| Item Number | Food Name | Large Store Retail Price (yuan) | Free Market Price (yuan) |
|---|---|---|---|
| 1 | Common carp (per jin) | **P102** ☐☐.☐ | **P56** ☐☐.☐ |
| 2 | Hair-tailed fish (per jin) | ☐☐.☐ | ☐☐.☐ |
| 2a | "Big head" spotted silver carp (per jin) | ☐☐.☐ | ☐☐.☐ |
| 3 | Fish, most commonly eaten (per jin) (specify: _______) | ☐☐.☐ | ☐☐.☐ |

**Table VIII. Bean Curd**

| Item Number | Food Name | Large Store Retail Price (yuan) | Free Market Price (yuan) |
|---|---|---|---|
| 1 | Bean curd, pressed (per jin) | **P103** ☐☐.☐ | **P64** ☐☐.☐ |
| 2 | Bean curd (per jin) | ☐☐.☐ | ☐☐.☐ |

**Table IX. Fuel**

| Item Number | Food Name | Retail Price (yuan) |
|---|---|---|
| 1 | Coal, honey-combed briquet (**per piece**) | **P104 [P106]** ☐☐.☐ |
| 2 | Coal lumps (per jin) | ☐☐.☐ |
| 3 | Coal powder (per jin) | ☐☐.☐ |
| 4 | Liquified natural gas (**per tank**) | ☐☐.☐ |
| 5 | Gasoline (**per liter**) | ☐☐.☐ |
| 6 | Kerosene (**per liter**) | ☐☐.☐ |
| 7 | Piped gas (**per cubic meter**) | ☐☐.☐ |
| 8 | Electricity (**per kilowatt hour**) | ☐☐.☐ |

**Table X. Other Products**

| Item Number | Food Name | Large Store Retail Price (yuan) | Free Market Price (yuan) |
|---|---|---|---|
| 1a | Hong Ta Shan (most expensive cigarettes) (**per pack—20 cigarettes**) | **P94** □□.□ | **P91** □□.□ |
| 2 | Marlboro cigarettes (**per pack—20 cigarettes**) | □□.□ | □□.□ |
| 2a | Double-Happiness cigarettes (from Wuhan) (**per pack—20 cigarettes**) | □□.□ | □□.□ |
| 1 | Local commonly smoked cigarettes (specify: _____) (**per pack—20 cigarettes**) | □□.□ | □□.□ |
| 3 | Local beer (**per bottle—640 ml**) | □□.□ | □□.□ |
| 4a | Luzhou aged alcohol (**per bottle—500 ml**) | □□.□ | □□.□ |
| 4 | Local liquor (**per bottle—500 ml**) | □□.□ | □□.□ |
| 6 | Cephalosporin (antibiotic) (**per 100 pills**) | □□.□ | □□.□ |
| 7 | Tagamet (antacid) (**per 100 pills**) | □□.□ | □□.□ |
| 8 | Capoten (hypertension drug) (**per 100 pills**) | □□.□ | □□.□ |
| 9 | Shuanghuanglian (Chinese traditional herbal medicine used for upper respiratory infections) (**per 100 pills**) | □□.□ | □□.□ |
| 0 | Venorutoni (cardiovascular drug) (**per 100 pills**) | □□.□ | □□.□ |
| 11 | Aspirin (**per 100 pills**) | □□.□ | □□.□ |
| 12 | Coca-Cola (**per can—355 ml**) | □□.□ | □□.□ |
| 13 | Jian Li Bao (soft drink with no caffeine) (**per can—355 ml**) | □□.□ | □□.□ |
