# Supplementary material for: Longitudinal measurement invariance in urbanization index of Chinese communities across 2000 and 2015: a Bayesian approximate measurement invariance approach
Source: BMC Public Health. 2021 Sep 10;21:1653. doi: 10.1186/s12889-021-11691-y (PMC8431910; doi:10.1186/s12889-021-11691-y)
Supplement: Supplementary file 4 — Additional file 4. [file 12889_2021_11691_MOESM4_ESM.pdf]

CHINA ECONOMIC, POPULATION, NUTRITION, AND HEALTH SURVEY

2011 COMMUNITY QUESTIONNAIRE

Province	21	Liaoning	23	Heilongjiang	32	Jiangsu	37	Shandong	41	Henan	_ _	T1
	42	Hubei	43	Hunan	45	Guangxi	52	Guizhou				

Urban Site: 1 | Rural Site: 2 | _ | T2

City: ___________________ | County: ________________ | _ | T3

City		County	
1	First city	1	First county
2	Second city	2	Second county
		3	Third county
		4	Fourth county

Neighborhood: ________________ | Village (Town): _______________ | _ _ | T4

Neighborhood		Village (Town)	
01	First [urban] neighborhood	01	County town neighborhood
02	Second [urban] neighborhood	02	First village
03	Third suburban village (neighborhood)	03	Second village
04	Fourth suburban village (neighborhood)	04	Third village
05	Fifth [urban] neighborhood	05	County town neighborhood
06	Sixth [urban] neighborhood	06	Fourth village
07	Seventh suburban village (neighborhood)	07	Fifth village
08	Eighth suburban village (neighborhood)	08	Sixth village
09	Ninth [urban] neighborhood	09	County town neighborhood
10	Tenth [urban] neighborhood	10	Seventh village
11	Eleventh suburban village (neighborhood)	11	Eighth village
12	Twelfth suburban village (neighborhood)	12	Ninth village

Person responded: _ _ _ _ Contact information:

Interview Date: _ _ _ _Year _ _Month _ _Day _ _ _ _ _ _ _ _ T7

Completion Evaluation: 1 Good 2 OK 3 Poor _ CO

Interviewer Name: ______________ Number: ______________ _ _ T6c

Supervisor Name: _______________ Number: ______________ _ _ T6d

One Community questionnaire should be completed for each village/neighborhood. Note that each community (village or neighborhood) is a government-designated administrative district, not a natural population cluster. The Community questionnaire includes the following sections:

Part A INFRASTRUCTURE, SERVICES AND ORGANIZATION

I Community Background (ask community head).. 1
II Demographics (ask community head and community accountant)................................. 2
III TV Channels (ask community head)... 3
IV Child Care Facilities and Schools (ask community head)... 5
V Large Stores, Supermarkets, Hypermarkets and Cooperatives: Presence, Location, Size and Impact on Other Food Retailers (ask community head).. 6
VI Free Markets: Presence, Location and Size (ask community head)................................. 8
VII Fast Food Restaurants & Chinese Restaurants (ask community head)............................ 10
VIII Recreational Facilities (ask community head).. 11
IX Other Facilities and Services (ask community head)... 12
X Medical Insurance (ask community health worker)... 14
XI Health Facilities (ask community health worker).. 14
XII Family Planning (ask community family planning head)... 17

Part B PRICES OF FOOD AND SPECIFIC LIVING MATERIAL
(ask community head or appropriate vendor/salesperson)

I Food Grains.. 19
II Cooking Oil and Sugar.. 19
III Vegetables and Fruits... 20
IV Meat and Poultry.. 20
V Fresh Milk.. 20
VI Preserved Milk Products... 20
VII Fish.. 21
VIII Bean Curd.. 21
IX Fuel.. 21
X Cigarette, Alcohol & Soft Drinks... 22
XI Medicines... 22

Part C GPS data collection table

I Nearest store, free market, restaurant, food stall, hospital, and drug store…………… 23
II Locations where food price available…………………………………………………… 24
III Household locations…………………………………………………………………….. 25

Part D Reasons household or household member did not participate

PART A: INFRASTRUCTURE, SERVICES AND ORGANIZATION

I. COMMUNITY BACKGROUND (ask community head)

*** From the official records, obtain the following statistics:**

1.	To which administrative district did this community belong in 2009?<br>1 city neighborhood<br>2 suburban neighborhood<br>3 town neighborhood<br>4 rural village	_	O1a
2.	Has the administrative district of this community changed since 2009?<br>0 no (skip to Question 5)<br>1 Yes<br>9 unknown (skip to Question 5)	_	O1b
3.	What is the current administrative district of this community?<br>1 city neighborhood<br>2 suburban neighborhood<br>3 town neighborhood<br>4 rural village<br>5 other (specify: _ _ _ _ _ _ _ _ _ _)	_	O1c
4.	In which year was the administrative district of the community changed?	_ _ _ _	O1d
5.	Has the geographic boundary of this community changed since 2009?<br>0 no (skip to question 7)<br>1 yes	_	O256
6.	In which year was the geographic boundary changed?	_ _ _ _	O257
7.	What is the population of the village/neighborhood?<br>* If "unknown," record -9999.	_ _ _ _ _	O0
8.	What was the area of the village/ neighborhood? (square km)<br>* If "unknown," record -99.99.	_ _ _._ _	O1
9.	How many households are in this village/neighborhood?	_ _ _ _	O0a
10.	What is the total population of this county/city?<br>* If the community is a town neighborhood/rural village, record the total population of the county. If the community is an urban/suburban neighborhood, record the total population of the city.	_ _ _ _ _ _ _ _ _	O270
11.	What is the total area of this county/city? (square km)<br>* If the community is a town neighborhood/rural village, record the total area of the county. If the community is an urban/suburban neighborhood, record the total area of the city.	_ _ _ _ _._ _	O271

II. DEMOGRAPHICS (ask community head and community accountant)

*** Ask Questions 2-3 about each occupation and record the answers in Table 1.**

Table 1. Common Occupations

1<br>Occupation	2<br>What is the daily wage for this occupation in the village/neighborhood? (yuan)<br>* If "unknown," record -99.		3<br>In addition to this wage, is free food provided?<br>0 no 1 yes 9 unknown	
Ordinary male worker	_ _ _	**O47a**	_	**O47b**
Ordinary female worker	_ _ _	**O47c**	_	**O47d**
Primary school teacher	_ _ _	**O272**	_	**O273**
Middle school teacher	_ _ _	**O274**	_	**O275**
Nanny hired by households	_ _ _	**O48**	_	**O48a**
Ordinary construction worker	_ _ _	**O49**	_	**O50**

4. What is the monthly income for a driver who is employed by a work unit and lives in this village/neighborhood? (yuan) _ _ _ _ O51

5 Is there an open trade area, an open city, or a special economic zone near this village/neighborhood (within two hours by bus)? _ O40
    0 no
    1 yes

6 What percentage of the work force in this village/neighborhood:
    (1) Is engaged mainly in agricultural activity (%) _ _ _ O42
    (2) Worked out of town for > 1 month last year (%) _ _ _ O43
    (3) Works in enterprises employing ≥ 20 people (%) _ _ _ O44
    (4) Works in enterprises employing < 20 people (%) _ _ _ O45

7 Are there any enterprises run by the village/neighborhood? _ O52
    0 no (skip to Question 11)
    1 yes

8 How many such enterprises are there? _ _ _ O53

9 What percentage of enterprises in this village/neighborhood is run by the village/neighborhood? (%) _ _ _ O63

10 How many private enterprises are in this village/neighborhood? _ _ _ O56a

11 Is there farm land in this village/neighborhood? _ O57
    0 no (skip to Question 13)
    1 yes

12 What percentage of the farm land is:
    (1) Irrigated (%) _ _ _ O57a
    (2) Irrigated by unified irrigation (%) _ _ _ O57b
    (3) Collectively plowed (%) _ _ _ O57c

13 How many of these businesses are currently operating in this village/neighborhood?

(1)	Fast food restaurants (e.g., McDonald's, Kentucky Fried Chicken)	_ _	O276
(2)	Other indoor restaurants	_ _	O60
(3)	Outdoor fixed food stalls	_ _	O61
(4)	Mobile food carts that sell cooked food(e.g., dumplings, steamed bread, pancakes)	_ _	O62
(5)	Bakeries and vendors that sell fried, twisted bread or other breakfast foods	_ _	O277
(6)	Bars and vendors that sell alcoholic beverages	_ _	O278
(7)	Ice cream parlors	_ _	O279
(8)	Fruit/vegetable stores and vendors (excluding seasonal vendors)	_ _	O280
(9)	Cafes (coffee or tea houses)	_ _	O281
(10)	Internet cafes	_ _	O282

III. TV CHANNVEL (ask community head)

***Ask questions 3-4 about each TV channel and record the answers in Table 2**

Table 2. TV Channels

1<br>Station Type	2<br>Channel Name	3<br>Is this channel available to residents in this village/neighborhood?<br>0 no 1 yes<br>* If "no," skip down to next item.<br>**O92**	4<br>In which year was this channel first available?<br>*If "unknown," record -999.<br>**O93**
1 Central TV	101 CCTV	_	_ _ _ _
2 Local TV	201 Province	_	_ _ _ _
	202 City	_	_ _ _ _
	203 County	_	_ _ _ _
3 Satellite TV	301 Beijing	_	_ _ _ _
	302 Tianjin	_	_ _ _ _
	303 Hebei	_	_ _ _ _
	304 Shanxi	_	_ _ _ _
	305 Inner Mongolia	_	_ _ _ _
	306 Liaoning	_	_ _ _ _
	307 Jilin	_	_ _ _ _
	308 Heilongjiang	_	_ _ _ _
	309 Shanghai	_	_ _ _ _
	310 Jiangsu	_	_ _ _ _
	311 Zhejiang	_	_ _ _ _

	312 Anhui	_	_ _ _ _
	313 Fujian	_	_ _ _ _
	314 Jiangxi	_	_ _ _ _
	315 Shandong	_	_ _ _ _
	316 Henan	_	_ _ _ _
	317 Hubei	_	_ _ _ _
	318 Hunan	_	_ _ _ _
	319 Guangdong	_	_ _ _ _
	320 Guangxi	_	_ _ _ _
	321 Hainan	_	_ _ _ _
	322 Sichun	_	_ _ _ _
	323 Chongqing	_	_ _ _ _
	324 Guizhou	_	_ _ _ _
	325 Yunnan	_	_ _ _ _
	326 Tibet	_	_ _ _ _
	327 Shaanxi	_	_ _ _ _
	328 Gansu	_	_ _ _ _
	329 Qinghai	_	_ _ _ _
	330 Ningxia	_	_ _ _ _
	331 Xinjiang	_	_ _ _ _
	332 Hong Kong	_	_ _ _ _
	334 China Entertainment TV	_	_ _ _ _
	333 Other (Specify:___________)	_	_ _ _ _
4 Cable TV	400 Cable TV	_	_ _ _ _

IV. CHILD CARE FACILITIES AND SCHOOLS (ask community head)
* Ask Questions 2-4 about each type of facility and record the answers in Table 3.

Table 3. Child Care Facilities

1<br>Age Group	2<br>Is there a child care facility for this age group in this village/neighborhood?<br>0 no 1 yes<br>* If "yes," skip to Question 4.<br>**O67a**	3<br>If "no," how far away is the nearest child care facility of this type? (km)<br>**O68a**	4<br>What is the average monthly fee for this age group? (yuan)<br>**O69a**
< 3 years	_	_ _._	_ _ _._
3-6 years	_	_ _._	_ _ _._

5. Is there a preschool for children managed by a primary school in this village/neighborhood? _ O76
   0 no (skip to Table 4)
   1 yes

6. What is the minimum age of children who can go to this school? (years) _ O76a

7. Is there a charge for a child to attend this preschool? _ O77
   0 no (skip to Table 4)
   1 yes

8. What is the fee per child per semester, excluding food? (yuan) _ _ _ _ _ O78a

*** Ask Questions 10-11 about each type of school and record the answers in Table 4**.

Table 4. Public Schools

9<br>School Type	10<br>Is there a school of this type in this village/neighborhood?<br>0 no 1 yes 9 unknown<br>* If "yes," skip down to next item.	11<br>If "no," how far away is the nearest school of this type? (km)<br>* If "unknown," record -99.9.
Primary school	_ **O79**	_ _ _._ **O80**
Lower middle school	_ **O81**	_ _ _._ **O82**
Upper middle school	_ **O83**	_ _ _._ **O84**
Vocational upper middle school or vocational technical school	_ **O85**	_ _ _._ **O86**
College	_ **O85a**	_ _ _._ **O86a**

V. LARGE STORES, SUPERMARKETS, HYPERMARKETS AND COOPERATIVES: PRESENCE, LOCATION, SIZE AND IMPACT ON OTHER FOOD RETAILERS (ask community head)

* A “hypermarket” is a very large store that sells products found in supermarkets as well as products commonly found in department stores.

*** Ask Questions 3-6 about each product and record the answers in Table 5.**

3. To what large store/supermarket/hypermarket/cooperative do residents in this village/neighborhood go most often to buy this product?
   * Record the store name in item 3 in Table 5.

4. What type of store is this?
   1 Western-owned supermarket/hypermarket(e.g., Metro, Makro, Carrefour, Wal-Mart, Auchan)
   2 Chinese-owned supermarket/hypermarket (e.g., Lianhua, Hualian, Huarun-Wanjia, Wumei)
   3 other Asian-owned supermarket/hypermarket(e.g., Park ’n Shop, Trust-Mart, Lotus, Acon/Jusco, Ito-Yokado)
   4 other large store or cooperative store
   5 other store
   9 never buy or no store available

5. Where is this large store/supermarket/hypermarket/cooperative?
   1 in this village/neighborhood
   2 in this city but a different neighborhood
   3 in another village/town/city
   9 never buy or no store available

6. How far away is this large store/supermarket/hypermarket/cooperative? (km)
   * If in this village/neighborhood, record 00.0. Otherwise, record the actual distance.

Table 5. Large Stores/Supermarkets/Hypermarkets/Cooperatives Where You Buy Food and Other Products

1<br>Item Number	2<br>Item Name	3<br>Store Name	4<br>Type<br>**O283**	5<br>Location<br>**O13a**	6<br>Distance (km)<br>**O14a**
1	Food grains		_	_	_ _._
2	Cooking oil		_	_	_ _._
3	Vegetables		_	_	_ _._
3a	Fruits		_	_	_ _._
4	Meat, poultry & eggs		_	_	_ _._
5	Fresh milk		_	_	_ _._
6	Preserved milk (canned milk, infant formula, etc.)		_	_	_ _._
7	Fish		_	_	_ _._
8	Bean curd		_	_	_ _._
11	Cigarettes		_	_	_ _._
12	Alcohol		_	_	_ _._
13	Soft drinks		_	_	_ _._

7. How many supermarkets or hypermarkets are within 5 kilometers from this village/neighborhood? _ _ O284

8. Where is the supermarket/hypermarket to which most residents in this village/neighborhood go to shop? _ O285

    1 in this village/neighborhood
    2 in this city but a different neighborhood
    3 in another village/town/city
    9 no supermarket/hypermarket available (skip to Question 15)

9. How far away is this supermarket/hypermarket? (km) _ _._ O286
   * If in this village/neighborhood, record 00.0. Otherwise, record the actual distance.

10. How big is the supermarket/hypermarket to which most residents of this village/neighborhood go to shop?
   (1) Total number of floors _ _ O319
   (2) Total area of all floors (square meters) _ _ _ _ _ O320

*** Ask Questions 11-13 about each product and record the answers in Table 6.**
Table 6. Supermarket/Hypermarket Food Retail Environment: Variety and Availability

11<br>Food item	12<br>How many different types of this food are available in this supermarket/hypermarket?	13<br>How much total shelf space is used for this food (square meters)?
Fresh fruit	_ _ _ **O321**	_ _ **O322**
Fresh vegetables	_ _ _ **O323**	_ _ **O324**
Snack foods, candy	_ _ _ **O325**	_ _ **O326**

14. Is this supermarket/hypermarket Western-owned, Chinese-owned, or owned by another Asian country (e.g., Thailand, Malaysia, Japan)? _ O287

    1 Western-owned (e.g., Metro, Makro, Carrefour, Wal-Mart, Auchan)
    2 Chinese-owned (e.g., Lianhua, Hualian, Huarun-Wanjia, Wumei)
    3 other-Asian-owned (e.g., Park ’n Shop, Trust-Mart, Lotus, Acon/Jusco, Ito-Yokado)
    9 unknown

15. Where is the Western-owned supermarket/hypermarket nearest this village/neighborhood? _ O288

    1 in this village/neighborhood
    2 in this city but a different neighborhood
    3 in another village/town/city
    9 no Western supermarket/hypermarket available (skip to Question 17)

16. How far away is this Western supermarket/hypermarket? (km) _ _._ O289
   * If in this village/neighborhood, record 00.0. Otherwise, record the actual distance.

17. Where is the Chinese-owned supermarket/hypermarket nearest this village/neighborhood? _ O290

    1 in this village/neighborhood
    2 in this city but a different neighborhood
    3 in another village/town/city
    9 no Chinese supermarket/hypermarket available (skip to Question 19)

18. How far away is this Chinese supermarket/hypermarket? (km) _ _._ O291
* If in this village/neighborhood, record 00.0. Otherwise, record the actual distance.

19. Where is the other-Asian-owned supermarket/hypermarket (Thai, Malaysian, Japanese) near this village/neighborhood? _ O292
   1 in this village/neighborhood
   2 in this city but a different neighborhood
   3 in another village/town/city
   9 no other-Asian supermarket/hypermarket available (skip to Question 21 in Table 7)

20. How far away is this other-Asian supermarket/hypermarket? (km) _ _._ O293
* If in this village/neighborhood, record 00.0. Otherwise, record the actual distance.

*** Ask Questions 21-23 about each product and record the answers in Table 7.**
Table 7. Nontraditional Foods Commonly Found in Supermarkets/Hypermarkets

21<br>Food item	22<br>Is this food commonly found in supermarkets/hypermarkets?<br>0 no 1 yes<br>9 no supermarket/hypermarket available	23<br>Is this food commonly found in free markets?<br>0 no 1 yes<br>9 no free market available
Non-sweetened instant cereals	_ **O296**	_ **O297**
Sweetened instant cereals	_ **O298**	_ **O299**
Potato chips or sticks	_ **O300**	_ **O301**
Chocolate or chocolate snack bars	_ **O302**	_ **O303**
Healthy snack foods (low in fat or sugar, according to label)	_ **O304**	_ **O305**
Cow’s milk (fresh)	_ **O306**	_ **O307**

24. During the past 2 years, how many supermarkets/hypermarkets within 5 kilometers have gone out of business? _ _ O294

25. During the past 2 years, how many new supermarkets/hypermarkets within 5 kilometers have opened? _ _ O295a

VI. FREE MARKETS AND GROCERY STORES: PRESENCE, LOCATION AND SIZE (ask community head) *** Ask Questions 3-7 about each product and record the answers in Table 8.**

3. To what free market do residents in this village/neighborhood go most often to buy this product?
* Record the market name in item 3 in Table 8. If the free market has no name, record its accurate address.

4. Where is this free market?
   1 in this village/neighborhood
   2 in this city but a different neighborhood
   3 in another village/town/city
   9 never buy or no market available

5. How far away is this free market? (km)
* If in this village/neighborhood, record 00.0. Otherwise, record the actual distance.

6. How many days per week is this free market open?

7. Do residents in this village/neighborhood purchase this item more often in this free market than in supermarkets/hypermarkets?
   0 no
   1 yes

Table 8. Free Markets Where You Buy Food and Other Products

1<br>Item<br>Number	2<br>Item Name	3<br>Free Market<br>Name	4<br>Location<br><br>**O16**	5<br>Distance<br>(km)<br><br>**O17**	6<br>Days/<br>Week<br><br>**O17a**	7<br>More<br>Often Purchased<br>Here?<br>**O308**
1	Food grains		_	_ _._	_	_
2	Cooking oil		_	_ _._	_	_
3	Vegetables		_	_ _._	_	_
3a	Fruits		_	_ _._	_	_
4	Meat, poultry and eggs		_	_ _._	_	_
5	Fresh milk		_	_ _._	_	_
6	Preserved milk (canned milk, infant formula, etc.)		_	_ _._	_	_
7	Fish		_	_ _._	_	_
8	Bean curd		_	_ _._	_	_
11	Cigarettes		_	_ _._	_	_
12	Alcohol		_	_ _._	_	_
13	Soft drinks		_	_ _._	_	_

8. How many free markets are within 5 kilometers from this village/neighborhood? _ _ O309a

9. Where is the free market nearest this village/neighborhood? _ O310
    1 in this village/neighborhood
    2 in this city but a different neighborhood
    3 in another village/town/city
    9 no free market available (skip to Question 12)

10. How far away is this free market? (km) _ _._ O311
    * If in this village/neighborhood, record 00.0. Otherwise, record the actual distance.

11. Is this free market open-air or enclosed? _ O312
    1 open-air
    2 enclosed

12. How many vendors are currently operating in the largest free market to which residents in this village/neighborhood go to shop? _ _ _ O313

13. If the following foods are available from supermarkets/hypermarkets, are residents in this village/neighborhood less likely to purchase them from free markets?

(1)	Food Grains	0 no	1 yes	_	O314
(2)	Fruits	0 no	1 yes	_	O315
(3)	Vegetables	0 no	1 yes	_	O316
(4)	Snack foods	0 no	1 yes	_	O317
(5)	Packaged[/instant] foods	0 no	1 yes	_	O318

14. How many grocery stores are within 5 kilometers from this village/neighborhood? _ _ O309b

15. Where is the grocery store nearest this village/neighborhood? _ O310a
   1 in this village/neighborhood
   2 in this city but a different neighborhood
   3 in another village/town/city
   9 no free market available (skip to next section)

16. How far away is this grocery store? (km) _ _._ O311a
   * If in this village/neighborhood, record 00.0. Otherwise, record the actual distance.

VII. FAST FOOD RESTAURANTS AND CHINESE RESTAURANTS (ask community head)

1. Are there any fast food restaurants, such as McDonald's or Kentucky Fried Chicken, near this village/neighborhood? _ O327
   0 no (skip to the next section)
   1 yes
   9 unknown (skip to the next section)

*** Ask Questions 3-7 about each restaurant and record the answers in Table 9.**

Table 9. Fast Food Restaurants

**2**<br>**Item Number**	**3**<br>**What is the name of this fast food restaurant?**	**4**<br>**Where is this restaurant?**<br>1 in this village/neighborhood<br>2 in this city but a different neighborhood<br>3 in another village/town/city<br>9 unknown or no restaurant of this type available<br>* If code 9 is used, skip down to next item.<br>**O248**	**5**<br>**How far away is this restaurant? (km)**<br>* If in this village/neighborhood, record 000.0. Otherwise, record the actual distance.<br>**O249**	**6**<br>**What is the price for a chicken burger ?**<br>**O350**	**7**<br>**What is the price for a regular cup of Coco-Cola?**<br>**O351**
1	McDonald's	_	_ _ _._	_ _ . _	_ _ . _
2	Kentucky Fried Chicken	_	_ _ _._	_ _ . _	_ _ . _
2a	Pizza Hut	_	_ _ _._	_ _ . _	_ _ . _
3	Other (specify: ___)	_	_ _ _._	_ _ . _	_ _ . _
4	Other (specify:___)	_	_ _ _._	_ _ . _	_ _ . _

8. How many Chinese restaurants are within 5 kilometers from this village/neighborhood? _ _ O352

9. Where is the Chinese restaurant nearest this village/neighborhood? _ O353
   1 in this village/neighborhood
   2 in this city but a different neighborhood
   3 in another village/town/city
   9 no Chinese restaurant available (skip to Question 13)

10. How far away is this Chinese restaurant? (km) _ _ . _ O354

11. What is the price of gongbao chicken in this restaurant? _ _ . _ O355a

12. What is the price of a large bottle of Coca-Cola in this restaurant (1.25 L/bottle)? _ _ . _ O355b

13. How many food stalls are within 5 kilometers from this village/neighborhood? _ _ O356

14. Where is the food stall nearest this village/neighborhood? _ O357
   1 in this village/neighborhood
   2 in this city but a different neighborhood
   3 in another village/town/city
   9 no food stall available (skip to next section)

15. How far away is this food stall? (km) _ _ . _ O358

16. What is the price of gongbao chicken in this food stall? _ _ . _ O359a

17. What is the price of a large bottle of Coca-Cola in this food stall(1.25 L/bottle)? _ _ . _ O359b

VIII. RECREATIONAL FACILITIES (ask community head)

1. Where is the gym or exercise center nearest this village/neighborhood? _ O250
   1 in this village/neighborhood
   2 in this city but a different neighborhood
   3 in another village/town/city
   9 no gym/exercise center available (skip to Question 3)

2. How far away is this gym/exercise center? (km) _ _ . _ O251
   * If in this village/neighborhood, record 00.0. Otherwise, record the actual distance.

3. Where is the park/public recreation place nearest this village/neighborhood? _ O252
   * Parks/public recreation places are spaces with or without facilities for children or adults to play or participate in sports/physical activities.
   1 in this village/neighborhood
   2 in this city but a different neighborhood
   3 in another village/town/city
   9 no park/public recreation place available (skip to Question 6)

4. How far away is this park/public recreation place? (km) _ _ . _ O253
   * If in this village/neighborhood, record 00.0. Otherwise, record the actual distance.

5. Does this park/public recreation place have the following facilities?

(1)	Soccer fields	0 no	1 yes	9 unknown	_	O328
(2)	Basketball courts	0 no	1 yes	9 unknown	_	O329
(3)	Volleyball, badminton or tennis courts	0 no	1 yes	9 unknown	_	O330
(4)	Running or walking trails	0 no	1 yes	9 unknown	_	O331
(5)	Ping pong tables	0 no	1 yes	9 unknown	_	O332
(6)	Other special sports facilities (specify:_ _ _ _)	0 no	1 yes	9 unknown	_	O333

6. Where is the playground nearest this village/neighborhood which is accessible to most residents? _ O334
* Playgrounds are spaces with facilities for children or adults to play or participate in sports/physical activities which are maintained by an institution, school, or government department but may have restricted use (e.g., employees of the institution or students of the school only).
   1 in this village/neighborhood
   2 in this city but a different neighborhood
   3 in another village/town/city
   9 no playground available (skip to the next section)
7. How far away is this playground? (km) _ _._ O335
* If in this village/neighborhood, record 00.0. Otherwise, record the actual distance.

8. Does this playground have the following facilities?

(1)	Soccer fields	0 no	1 yes	9 unknown	_	O336
(2)	Basketball courts	0 no	1 yes	9 unknown	_	O337
(3)	Volleyball, badminton or tennis courts	0 no	1 yes	9 unknown	_	O338
(4)	Running or walking trails	0 no	1 yes	9 unknown	_	O339
(5)	Ping pong tables	0 no	1 yes	9 unknown	_	O340
(6)	Other special sports facilities (specify:_ _ _ _ _)	0 no	1 yes	9 unknown	_	O341

IX. OTHER FACILITIES AND SERVICES (ask community head)

1. Are there fewer residents who bike to work now than in 2006 in this village/neighborhood? _ O254
   0 no (skip to Question 3)
   1 yes
2. Why did they stop biking to work?

(1)	Environmental pollution	0 no	1 yes	9 unknown	_	O255_1
(2)	Too much traffic or traffic too dangerous	0 no	1 yes	9 unknown	_	O255_2
(3)	Fewer bicycle lanes now	0 no	1 yes	9 unknown	_	O255_3
(3a)	Drive car/take taxi now	0 no	1 yes	9 unknown	_	O255_3a
(3b)	Public transportation improved	0 no	1 yes	9 unknown	_	O255_3b
(4)	Other (specify: _ _ _ _ _ _ _ _ _ _ _)	0 no	1 yes	9 unknown	_	O255_4

3. What is the most common characteristic of the roads in or around this village/neighborhood? _ O23
   1 dirt
   2 stone, gravel, or mixed material (skip to Question 6)
   3 paved road (skip to Question 6)
4. Are there any stone, gravel, or paved roads? _ O23a
   0 no
   1 yes (skip to Question 6)
5. How far away is the nearest stone, gravel, or paved road? (km) _ _._ O24

6. Are the following services available in this village/neighborhood?

(1)	Convenient telegraph service	0 no	1 yes	9 unknown	_	O25
(2)	Convenient telephone service	0 no	1 yes	9 unknown	_	O26
(3)	Cell phone service	0 no	1 yes	9 unknown	_	O342
(4)	Convenient internet service	0 no	1 yes	9 unknown	_	O343
(5)	Convenient fax service	0 no	1 yes	9 unknown	_	O26a
(6)	Postal service	0 no	1 yes	9 unknown	_	O27
(7)	Provincial daily newspaper received on the day it is published	0 no	1 yes	9 unknown	_	O28
(8)	Convenient movies	0 no	1 yes	9 unknown	_	O29

7. Is electricity available in this village/neighborhood? _ O30
   0 no (skip to Question 10)
   1 Yes

8. When electricity is supplied, how many hours per day, on the average, is electricity available? (hours) _ _ O31

9. On the average, how many days per week is the electricity cut off? _ O32

10. Is there a bus stop (or long distance bus stop) in this village/neighborhood? _ O33
    0 No
    1 yes (skip to Question 13)

11. How far away is the nearest bus stop? (km) _ _._ O34

12. How often does the bus serve this bus stop? (minutes) _ _ _ O344

13. Is this village/neighborhood near a train station? _ O35
    0 no
    1 yes (skip to Question 15)

14. How far away is the nearest train station? (km) _ _ _._ O36

15. Are public baths available in this village/neighborhood? _ O37
    0 no
    1 yes (skip to Question 17)

16. How far away is the nearest public bath? (km) _ _._ O38

17. Is this village/neighborhood near a navigable river? _ O39
    0 no
    1 yes

X. MEDICAL INSURANCE (ask community health worker)
*** Ask Questions 3-4 about each type of medical insurance and record the answers in Table 10.**

Table 10. Medical Insurance

1<br>Item Number	2<br>Insurance Type	3<br>Does this village/ neighborhood have this type of medical insurance?<br>0 no 1 yes 9 unknown<br>* If "no" or "unknown," skip down to next item.<br>**O8cb**	4<br>In which year was this insurance first available?<br>* If "unknown," record -999.<br>**O8cc**
0	Commercial medical insurance	_	_ _ _ _
1	Free (or Government) Medical Insurance	_	_ _ _ _
2	Urban employee basic medical insurance	_	_ _ _ _
3	Urban resident basic medical insurance	_	_ _ _ _
4	Cooperative insurance	_	_ _ _ _
8	Other (specify: _ _ _ _ _ _ _ _ _)	_	_ _ _ _

XI. HEALTH FACILITIES (ask community health worker)
*** Ask Questions 2-10 about each health facility and record the answers in Table 11.**

2. When residents in this village/neighborhood need health services, what health facilities can they use?
   * Record the name of each facility in item 2 in Table 11.

3. What type of facility is this?
   - 01 village clinic
   - 02 private clinic
   - 03 work unit clinic
   - 04 other clinic
   - 05 town family planning service
   - 06 town hospital
   - 07 county maternal and child hospital
   - 08 county hospital
   - 09 city maternal and child hospital
   - 10 city hospital
   - 11 worker's hospital
   - 12 other hospital
   - 13 drug store
   - 15 other (specify: _ _ _ _ _ _ _ _ _ _ _)
   - \- 9 unknown

4. Where is this facility?
   - 1 in this village/neighborhood
   - 2 in this city but a different neighborhood
   - 3 in another village/town/city

5. How far away is this facility? (km)
   * If in this village/neighborhood, record 000.0. Otherwise, record the actual distance.

6. On the average, how many hours per week is this facility open for business?
7. How many doctors does this facility have?
8. How many hospital beds does this facility have?
9. How much is the registration fee at this facility? (yuan)
10. What is the treatment fee for a common cold in this facility? (yuan)

*** If respondent does not report at least 1 clinic, go back to Question 2 and ask about the most commonly used clinic. If respondent does not report at least 1 hospital, go back to Question 2 and ask about the most commonly used hospital.**

Table 11. Health Facilities

1<br>Facility Number	2<br>Facility Name	3<br>Facility Type<br>**O19b**	4<br>Location<br>**O20**	5<br>Distance (km)<br>**O21**	6<br>Busines s Hours (hrs/wk)<br>**O21a**	7<br>Number of Doctors<br>**O21b**	8<br>Number of Hospital Beds<br>**O21c**	9<br>Registration Fee (yuan)<br>**O21d**	10<br>Cold Treatment Fee (yuan)<br>**O21f**
1		_ _	_	_ _ _._	_ _ _	_ _ _ _	_ _ _ _	_ _._	_ _._
2		_ _	_	_ _ _._	_ _ _	_ _ _ _	_ _ _ _	_ _._	_ _._
3		_ _	_	_ _ _._	_ _ _	_ _ _ _	_ _ _ _	_ _._	_ _._
4		_ _	_	_ _ _._	_ _ _	_ _ _ _	_ _ _ _	_ _._	_ _._
5		_ _	_	_ _ _._	_ _ _	_ _ _ _	_ _ _ _	_ _._	_ _._

11. Have any new clinics or hospitals opened in this village/neighborhood since 2009? _ O21e
    0 no (skip to Question 20)
    1 yes

*** Ask Questions 13-19 about each new health facility and record the answers in Table 12.**

13. What is the name of this facility?
    *** Record the name of each facility in item 13 of Table 12**.

14 What type of facility is this?

01	village clinic	09	city maternal and child hospital
02	private clinic	10	city hospital
03	work unit clinic	11	worker's hospital
04	other clinic	12	other hospital
05	town family planning service	13	drug store
06	town hospital	15	other (specify: _ _ _ _ _ _ _ _ _ _)
07	county maternal and child hospital	- 9	unknown
08	county hospital		

15. Approximately when did this facility open for business? (year)

16. On the average, how many hours per week is this facility open for business?

17. How many doctors does this facility have?

18. How many hospital beds does this facility have?

19. How much is the registration fee at this facility? (yuan)

Table 12. Newly Opened Health Facilities

12 Facility Number	13 Facility Name	14 Facility Type **O231a**	15 Year Opened **O232**	16 Business Hours (hrs/wk) **O233**	17 Number of Doctors **O234**	18 Number of Hospital Beds **O235**	19 Registration Fee (yuan) **O236**
1		_ _	_ _ _ _	_ _ _	_ _ _ _	_ _ _ _	_ _._
2		_ _	_ _ _ _	_ _ _	_ _ _ _	_ _ _ _	_ _._
3		_ _	_ _ _ _	_ _ _	_ _ _ _	_ _ _ _	_ _._
4		_ _	_ _ _ _	_ _ _	_ _ _ _	_ _ _ _	_ _._

20 Did any clinic or hospital that residents from this village/neighborhood used regularly go out of business, or was any clinic/hospital replaced by another type of health facility since 2006? _ O236a

0 no (skip to the next section)
1 yes

*** Ask Questions 22-28 about each health facility and record the answers in Table 13.**

22. What was the name of this facility?
* Record the name of each facility in item 22 in Table 13.

23. What type of facility was this?

01 village clinic
02 private clinic
03 work unit clinic
04 other clinic
05 town family planning service
06 town hospital
07 county maternal and child hospital
08 county hospital
09 city maternal and child hospital
10 city hospital
11 worker's hospital
12 other hospital
13 drug store
15 other (specify: _ _ _ _ _ _ _ _ _ _ _)
- 9 unknown

24. Was this facility replaced by another type of facility?

0 no (skip to Question 26)
1 yes

25. Type of facility after replacement?

01	village clinic	09	city maternal and child hospital
02	private clinic	10	city hospital
03	work unit clinic	11	worker's hospital
04	other clinic	12	other hospital
05	town family planning service	13	drug store
06	town hospital	15	other (specify: _ _ _ _ _ _ _ _ _ _ _)
07	county maternal and child hospital	- 9	unknown
08	county hospital		

26. Where was this facility?
    1 in this village/neighborhood
    2 in this city/town but a different neighborhood
    3 in another village/town/city

27. How far away was this facility? (km)
    * If in this village/neighborhood, record 000.0. Otherwise, record the actual distance.

28. In which year did this facility go out of business?

Table 13. Health Facilities Closed or Replaced

21 Facility Number	22 Facility Name	23 Facility Type O238a	24 Replaced? O239	25 Facility Type After Replacement O240a	26 Location O241	27 Distance (km) O242	28 Year Went Out of Business O243
1		_ _	_	_ _	_	_ _ _._	_ _ _ _
2		_ _	_	_ _	_	_ _ _._	_ _ _ _
3		_ _	_	_ _	_	_ _ _._	_ _ _ _

XII. FAMILY PLANNING (ask community family planning head)

1. What percentage of the population in this village/neighborhood is from each of the following ethnic groups (nationalities)?
   * Percentages must add up to 100%.

(1)	Han (if 100%, [record 100, then] skip to Question 4)	_ _ _	R6j_1
(2)	Miao	_ _ _	R6j_6
(3)	Buyi	_ _ _	R6j_9
(4)	Man	_ _ _	R6j_11
(5)	Tujia	_ _ _	R6j_15
(6)	Other (specify: _ _ _ _ _ _ _ _ _ _ _)	_ _ _	R6j_20

2. Is the family planning policy the same for minorities as it is for Han nationality? _ R6b
   0 no
   1 yes

3.	Are minority couples in this village/neighborhood allowed to have two children?				_	R6k
	0 no					
	1 yes, but only if both the husband and wife are minorities					
	2 yes, as long as <u>either</u> the husband <u>or</u> wife is a minority					
4.	Are all couples in this village/neighborhood allowed to have more than two children?				_	R6i
	0 no					
	1 yes (skip to Question 7)					
5.	Are all couples in this village/neighborhood allowed to have two children?				_	R6
	0 no					
	1 yes (skip to Question 7)					
6.	Are couples of Han nationality allowed to have two children if:					
	(1) Their first child is a girl	0 no	1 yes	9 unknown	_	R1
	(2) Each parent is an only child	0 no	1 yes	9 unknown	_	R3
	(3) Both parents have certain special occupations	0 no	1 yes	9 unknown	_	R4
	(4) There are other exceptions (specify:_ _ _ _ _ _ _ _)	0 no	1 yes	9 unknown	_	R5d
7.	Have the local cadres implemented the family planning responsibility system?				_	R20d
	0 no					
	1 yes, connected with economic rewards					
	2 yes, not connected with economic rewards					
8.	Do couples receive a subsidy if they have only one child?				_	R16
	0 no					
	1 yes					
9.	Do one-child families receive child health care subsidies?				_	R33
	0 no					
	1 yes					

PART B: PRICES OF FOOD AND SPECIFIC LIVING MATERIAL
(ask community head or appropriate vendor/salesperson)
*** Obtain the following prices at the time of interview. If the village/neighborhood doesn't have the item or the price is "unknown," record -9.9.**

Table I. Food Grains

Item Number	Food Name	Large Store Retail Price (yuan) **P96**	Free Market Price (yuan) **P8**
1	Very high quality rice (per jin)	_ _._	_ _._
2	Rice, most commonly eaten (per jin)	_ _._	_ _._
3	Bleached flour (per jin)	_ _._	_ _._
4	Unbleached flour (per jin)	_ _._	_ _._
5	Noodles made of bleached flour (per jin)	_ _._	_ _._
6	Noodles made of unbleached flour (per jin)	_ _._	_ _._
7	Corn flour (per jin)	_ _._	_ _._
8	Millet (per jin)	_ _._	_ _._
9	Sorghum (per jin)	_ _._	_ _._

Table II. Cooking Oil and Sugar

Item Number	Food Name	Large Store Retail Price (yuan) **P97**	Free Market Price (yuan) **P16**
1a	Rape seed oil (cheapest, per jin)	_ _._	_ _._
1	Rape seed oil (most expensive, per jin)	_ _._	_ _._
2a	Soybean oil (cheapest, per jin)	_ _._	_ _._
2	Soybean oil (most expensive, per jin)	_ _._	_ _._
3a	Peanut oil (cheapest, per jin)	_ _._	_ _._
3	Peanut oil (most expensive, per jin)	_ _._	_ _._
4	Cottonseed oil (per jin)	_ _._	_ _._
5	Tea oil (per jin)	_ _._	_ _._
6	White sugar (per jin)	_ _._	_ _._
7	Eggs (per jin)	_ _._	_ _._
8	Soy sauce, most commonly used (per jin)	_ _._	_ _._
9	Vinegar, most commonly used (per jin)	_ _._	_ _._
10a	Refined oil (cheapest, per jin)	_ _._	_ _._
10	Refined oil (most expensive, per jin)	_ _._	_ _._

Table III. Vegetables and Fruits

Item Number	Food Name	Large Store Retail Price (yuan) **P98**	Free Market Price (yuan) **P24**
1a	Dark green leafy vegetables (rape) (cheapest, per jin)	_ _._	_ _._
1	Dark green leafy vegetables (rape) (most expensive, per jin)	_ _._	_ _._
2a	Cabbage (cheapest, per jin)	_ _._	_ _._
2	Cabbage (most expensive, per jin)	_ _._	_ _._
3a	Vegetable, most commonly eaten (cheapest, per jin) (specify:_ _ _ _ _ _ _ _)	_ _._ (Not in Chinese version)	_ _._
3	Vegetable, most commonly eaten (most expensive, per jin) (specify:_ _ _ _ _ _ _ _)	_ _._	_ _._
4a	Apple (cheapest, per jin)	_ _._	_ _._
4	Apple (most expensive, per jin)	_ _._	_ _._
5a	Orange (cheapest, per jin)	_ _._	_ _._
5	Orange (most expensive, per jin)	_ _._	_ _._

Table IV. Meat and Poultry

Item Number	Food Name	Large Store Retail Price (yuan) **P99**	Free Market Price (yuan) **P32**
1	Pork, fatty and lean (per jin)	_ _._	_ _._
2	Pork, lean (per jin)	_ _._	_ _._
3	Live chicken (per jin)	_ _._	_ _._
4	Chicken, whole, cleaned (per jin)	_ _._	_ _._
5	Beef (per jin)	_ _._	_ _._
6	Mutton (per jin)	_ _._	_ _._

Table V. Milk

Item Number	Food Name	Large Store Retail Price (yuan) **P100**	Free Market Price (yuan) **P40**
1a	Fresh whole milk (international , 2**50 ml/package**)	_ _._	_ _._
1	Fresh whole milk (domestic, 2**50 ml/package**)	_ _._	_ _._
2	Ultra-high temperature processed milk (international, **250 ml/package**)	_ _._	_ _._
3	Ultra-high temperature processed milk (domestic, **250 ml/package**)	_ _._	_ _._

Table VI. Preserved Milk Products

Item Number	Food Name	Large Store Retail Price (yuan)<br>**P101**	Free Market Price (yuan)<br>**P48**
2a	Whole, powdered (international, per jin)	_ _._	_ _._
2	Whole, powdered (domestic, per jin)	_ _._	_ _._
3a	Substitute formula, soy or rice (international, per jin)	_ _._	_ _._
3	Substitute formula, soy or rice (domestic, per jin)	_ _._	_ _._
4a	Infant formula (international, per jin)	_ _._	_ _._
4	Infant formula (domestic, per jin)	_ _._	_ _._

Table VII. Fish

Item Number	Food Name	Large Store Retail Price (yuan)<br>**P102**	Free Market Price (yuan)<br>**P56**
1	Common carp (per jin)	_ _._	_ _._
2	Hair-tailed fish (per jin)	_ _._	_ _._
2a	"Big head" spotted silver carp (per jin)	_ _._	_ _._
3	Fish, most commonly eaten (per jin) (specify: _ _ _ _ _ _)	_ _._	_ _._

Table VIII. Bean Curd

Item Number	Food Name	Large Store Retail Price (yuan)<br>**P103**	Free Market Price (yuan)<br>**P64**
1	Bean curd, pressed (per jin)	_ _._	_ _._
2	Bean curd (per jin)	_ _._	_ _._

Table IX. Fuel

Item Number	Food Name	Retail Price (yuan)<br>**P104**
1	Coal, honey-combed briquet (**per piece**)	_ _._
2	Coal lumps (per jin)	_ _._
3	Coal powder (per jin)	_ _._
4	Liquified natural gas (**per tank**)	_ _._
5	Gasoline (**per liter**)	_ _._
6	Kerosene (**per liter**)	_ _._
7	Piped gas (**per cubic meter**)	_ _._
8	Electricity (**per kilowatt hour**)	_ _._

Table X. Cigarette, Alcohol & Soft Drinks

Item Number	Food Name	Large Store Retail Price (yuan) **P94**	Free Market Price (yuan) **P91**
14	Most expensive cigarettes (20 cigarettes/package)	_ _._	_ _._
1a	Hong Ta Shan (**20 cigarettes/package**)	_ _._	_ _._
2	Marlboro cigarettes (**20 cigarettes/package**)	_ _._	_ _._
1	Local commonly smoked cigarettes (specify: _ _ _ _ _) (**20 cigarettes/package**)	_ _._	_ _._
3	Local beer (**640 ml/package**)	_ _._	_ _._
15	Maotai (53% alcohol, 500 ml/bottle)	_ _._	_ _._
4a	Luzhou aged alcohol (**52% alcohol, 500 ml/bottle**)	_ _._	_ _._
4	Local liquor (**500 ml/bottle**)	_ _._	_ _._
12	Coca-Cola (**355 ml/can**)	_ _._	_ _._
13	Chinese soft drink (soft drink with no caffeine) (domestic, similar to coca-cola, **355 ml/can**)	_ _._	_ _._

Table XI. Medicines

Item Number	Food Name	Hospital (yuan) **P106**	How many pills/package (hospital) **P106a**	Drug Store (yuan) **P107**	How many pills/package (drugstore) **P107a**
6	Cephalosporin (antibiotic) (**per package**)	_ _._	_ _ _	_ _._	_ _ _
7	Tagamet (antacid) (**per package**)	_ _._	_ _ _	_ _._	_ _ _
8	Capoten (hypertension drug) (**per package**)	_ _._	_ _ _	_ _._	_ _ _
9	Shuanghuanglian (Chinese traditional herbal medicine used for upper respiratory infections) (**per package**)	_ _._	_ _ _	_ _._	_ _ _
10	Venorutoni (cardiovascular drug) (**per package**)	_ _._	_ _ _	_ _._	_ _ _
11	Aspirin (**per package**)	_ _._	_ _ _	_ _._	_ _ _

Part C: GPS data collection table

GPS ID: _ _ X0

Table I: Where is the nearest store, free market, restaurant, and food stall within 5 kilometers from this village/neighborhood? Where is the nearest hospital and drug store? (1st digit of XY_CODE is GPS Community Code for this GPS Receiver, 1-5)

XY_CODE	Items	Name	Latitude (N/S) X1	Longitude (W/E) X2	Offset, distance, and direction if more than 30 meters away
_ 100	Community center		_ _._ _ _ _ _	_ _ _._ _ _ _ _	
_ 101	Grocery store		_ _._ _ _ _ _	_ _ _._ _ _ _ _	
_ 102	Free market		_ _._ _ _ _ _	_ _ _._ _ _ _ _	
_ 103	Fast food restaurant	McDonald's	_ _._ _ _ _ _	_ _ _._ _ _ _ _	
_ 104		KFC	_ _._ _ _ _ _	_ _ _._ _ _ _ _	
_ 105		Pizza Hut	_ _._ _ _ _ _	_ _ _._ _ _ _ _	
_ 106		Other fast food restaurant	_ _._ _ _ _ _	_ _ _._ _ _ _ _	
_ 107	Chinese restaurant		_ _._ _ _ _ _	_ _ _._ _ _ _ _	
_ 108	Food Stall		_ _._ _ _ _ _	_ _ _._ _ _ _ _	
_ 109	Hospital		_ _._ _ _ _ _	_ _ _._ _ _ _ _	
_ 110	Drug store		_ _._ _ _ _ _	_ _ _._ _ _ _ _	

Table II: Where do you get the price for the items below?

XY_CODE	Availability	Grain<br><br>0 N 1 Y	Cooking oil<br>0 N 1 Y	Vegetable & fruit<br>0 N 1 Y	Meat & poultry<br>0 N 1 Y	Fresh milk<br>0 N 1 Y	Preserved milk<br>0 N 1 Y	Fish<br><br>0 N 1 Y	Bean curd<br>0 N 1 Y	Cigarette, alcohol, & soft drinks<br>0 No 1 Yes	Remarks
_ 111	Store #1<br>Lat: _ _ . _ _ _ _ _ X1<br>Lon: _ _ _ . _ _ _ _ _ X2	X3<br>_	X4<br>_	X5<br>_	X6<br>_	X7<br>_	X8<br>_	X9<br>_	X10<br>_	X11<br>_	
_ 112	Store #2<br>Lat: _ _ . _ _ _ _ _ X1<br>Lon: _ _ _ . _ _ _ _ _ X2	_	_	_	_	_	_	_	_	_	
_ 113	Market#1<br>Lat: _ _ . _ _ _ _ _ X1<br>Lon: _ _ _ . _ _ _ _ _ X2	_	_	_	_	_	_	_	_	_	
_ 114	Market#2<br>Lat: _ _ . _ _ _ _ _ X1<br>Lon: _ _ _ . _ _ _ _ _ X2	_	_	_	_	_	_	_	_	_	

Table III: Household locations

XY_HH	Latitude (N/S)<br>X1	Longitude<br>(W/E)<br>X2	Offset, distance, and direction if more than 30 meters away
2 _ _ _ _	_ _._ _ _ _ _ _	_ _ _._ _ _ _ _ _	
2 _ _ _ _	_ _._ _ _ _ _ _	_ _ _._ _ _ _ _ _	
2 _ _ _ _	_ _._ _ _ _ _ _	_ _ _._ _ _ _ _ _	
2 _ _ _ _	_ _._ _ _ _ _ _	_ _ _._ _ _ _ _ _	
2 _ _ _ _	_ _._ _ _ _ _ _	_ _ _._ _ _ _ _ _	
2 _ _ _ _	_ _._ _ _ _ _ _	_ _ _._ _ _ _ _ _	
2 _ _ _ _	_ _._ _ _ _ _ _	_ _ _._ _ _ _ _ _	
2 _ _ _ _	_ _._ _ _ _ _ _	_ _ _._ _ _ _ _ _	
2 _ _ _ _	_ _._ _ _ _ _ _	_ _ _._ _ _ _ _ _	
2 _ _ _ _	_ _._ _ _ _ _ _	_ _ _._ _ _ _ _ _	
2 _ _ _ _	_ _._ _ _ _ _ _	_ _ _._ _ _ _ _ _	
2 _ _ _ _	_ _._ _ _ _ _ _	_ _ _._ _ _ _ _ _	
2 _ _ _ _	_ _._ _ _ _ _ _	_ _ _._ _ _ _ _ _	
2 _ _ _ _	_ _._ _ _ _ _ _	_ _ _._ _ _ _ _ _	
2 _ _ _ _	_ _._ _ _ _ _ _	_ _ _._ _ _ _ _ _	
2 _ _ _ _	_ _._ _ _ _ _ _	_ _ _._ _ _ _ _ _	
2 _ _ _ _	_ _._ _ _ _ _ _	_ _ _._ _ _ _ _ _	
2 _ _ _ _	_ _._ _ _ _ _ _	_ _ _._ _ _ _ _ _	
2 _ _ _ _	_ _._ _ _ _ _ _	_ _ _._ _ _ _ _ _	
2 _ _ _ _	_ _._ _ _ _ _ _	_ _ _._ _ _ _ _ _	
2 _ _ _ _	_ _._ _ _ _ _ _	_ _ _._ _ _ _ _ _	
2 _ _ _ _	_ _._ _ _ _ _ _	_ _ _._ _ _ _ _ _	
2 _ _ _ _	_ _._ _ _ _ _ _	_ _ _._ _ _ _ _ _	
2 _ _ _ _	_ _._ _ _ _ _ _	_ _ _._ _ _ _ _ _	
2 _ _ _ _	_ _._ _ _ _ _ _	_ _ _._ _ _ _ _ _	
2 _ _ _ _	_ _._ _ _ _ _ _	_ _ _._ _ _ _ _ _	
2 _ _ _ _	_ _._ _ _ _ _ _	_ _ _._ _ _ _ _ _	
2 _ _ _ _	_ _._ _ _ _ _ _	_ _ _._ _ _ _ _ _	
2 _ _ _ _	_ _._ _ _ _ _ _	_ _ _._ _ _ _ _ _	
2 _ _ _ _	_ _._ _ _ _ _ _	_ _ _._ _ _ _ _ _	

XY_HH – 5-digit Household ID (2XTTT)
2 = 1-Digit Code for Household GPS Data
X = 1-digit Community GPS Code for this GPS Receiver (1-5)
TTT = 3-digit Household ID (T5)

Part D Reasons household or household member did not participate

Community ID: _ _ Province/District/City (T1) _ Survey Site (T2) _City/county/district (T3) _ _ _ Neighborhood/Village (T4)

**1 Househol d**	**2 Participate d?** 0 no 1 yes * if yes, skip to Q4	**3 Why did not participate** 1 moved out of comm. 2 sought employment elsewhere 3 rejected 4 other *skip to next household	**4 Line number**	**5 Name**	**6 Age (y)**	**7 Gender** 1 male 2 female	**8 Participate d?** 0 no 1 yes * if yes, skip to Q10	**9 Why did not participate** 1 moved out 2 deceased 3 not at home 4 gone to school 5 military services 6 sought employment elsewhere 7 gone abroad 8 rejected 9 other	**10 Data collected: 0 no 1 yes**		
									**Individu al Survey**	**PE**	**Diet**
**T5**	**Y1**	**Y2**	**AA1**		**Y3**	**Y4**	**Y5**	**Y6**	**Y7**	**Y8**	**Y9**
_ _ _	_	_	_ _ _		_ _ _	_	_	_	_	_	_
_ _ _	_	_	_ _ _		_ _ _	_	_	_	_	_	_
_ _ _	_	_	_ _ _		_ _ _	_	_	_	_	_	_
_ _ _	_	_	_ _ _		_ _ _	_	_	_	_	_	_
_ _ _	_	_	_ _ _		_ _ _	_	_	_	_	_	_
_ _ _	_	_	_ _ _		_ _ _	_	_	_	_	_	_
_ _ _	_	_	_ _ _		_ _ _	_	_	_	_	_	_
_ _ _	_	_	_ _ _		_ _ _	_	_	_	_	_	_
